# Tocilizumab for patients with COVID-19 pneumonia. The single-arm TOCIVID-19 prospective trial

**DOI:** 10.1186/s12967-020-02573-9

**Published:** 2020-10-21

**Authors:** Francesco Perrone, Maria Carmela Piccirillo, Paolo Antonio Ascierto, Carlo Salvarani, Roberto Parrella, Anna Maria Marata, Patrizia Popoli, Laurenzia Ferraris, Massimiliano M. Marrocco-Trischitta, Diego Ripamonti, Francesca Binda, Paolo Bonfanti, Nicola Squillace, Francesco Castelli, Maria Lorenza Muiesan, Miriam Lichtner, Carlo Calzetti, Nicola Duccio Salerno, Luigi Atripaldi, Marco Cascella, Massimo Costantini, Giovanni Dolci, Nicola Cosimo Facciolongo, Fiorentino Fraganza, Marco Massari, Vincenzo Montesarchio, Cristina Mussini, Emanuele Alberto Negri, Gerardo Botti, Claudia Cardone, Piera Gargiulo, Adriano Gravina, Clorinda Schettino, Laura Arenare, Paolo Chiodini, Ciro Gallo, the TOCIVID-19 investigators, Italy, Francesco Perrone, Maria Carmela Piccirillo, Clorinda Schettino, Adriano Gravina, Piera Gargiulo, Claudia Cardone, Laura Arenare, Paolo Antonio Ascierto, Maria Grazia Vitale, Claudia Trojaniello, Marco Palla, Attilio Antonio Montano Bianchi, Gerardo Botti, Gianfranco De Feo, Leonardo Miscio, Ciro Gallo, Paolo Chiodini, Laurenzia Ferraris, Massimiliano M. Marrocco-Trischitta, Marco Froldi, Lorenzo Menicanti, Maria Teresa Cuppone, Giulia Gobbo, Chiara Baldessari, Vincenzo Valenti, Serenella Castelvecchio, Federica Poli, Francesca Giacomazzi, Rosangela Piccinni, Maria Laura Annnunziata, Andrea Biondi, Cecilia Bussolari, Manuel Mazzoleni, Andrea Giachi, Annalisa Filtz, Arianna Manini, Enrico Poletti, Federico Masserini, Francesco Conforti, Gianfranco Gaudiano, Vittoria Favero, Alice Moroni, Tommaso Viva, Fabiana Fancoli, Davide Ferrari, Dario Niro, Marco Resta, Andrea Ballotta, Marco Dei Poli, Marco Ranucci, Diego Ripamonti, Francesca Binda, Alessandra Tebaldi, Giuseppe Gritti, Luisa Pasulo, Leonardo Gaglio, Roberto Del Fabbro, Leonardo Alborghetti, Paolo Bonfanti, Nicola Squillace, Giulia Giustinetti, Paola Columpsi, Marina Cazzaniga, Serena Capici, Luca Sala, Riccardo Di Sciacca, Giacomo Mosca, Maria Rosa Pirozzi, Francesco Castelli, Maria Lorenza Muiesan, Franco Franceschini, Aldo Roccaro, Massimo Salvetti, Anna Paini, Luciano Corda, Chiara Ricci, Lina Tomasoni, Paola Nasta, Silvia Lorenzotti, Silvia Odolini, Emanuele Focà, Eugenia Quiros Roldan, Marco Metra, Stefano Magrini, Paolo Borghetti, Nicola Latronico, Simone Piva, Matteo Filippini, Francesco Zuccalà, Sergio Cattaneo, Francesco Scolari, Nicola Bossini, Mario Gaggiotti, Martina Properzi, Miriam Lichtner, Cosmo Del Borgo, Raffaella Marocco, Valeria Belvisi, Tiziana Tieghi, Margherita De Masi, Paola Zuccalà, Paolo Fabietti, Angelo Vetica, Vito Sante Mercurio, Anna Carraro, Laura Fondaco, Blerta Kertusha, Ambrogio Curtolo, Emanuela Del Giudice, Riccardo Lubrano, Maria Gioconda Zotti, Antonella Puorto, Marcello Ciuffreda, Antonella Sarni, Gabriella Monteforte, Domenico Romeo, Emanuela Viola, Carla Damiani, Antonietta Barone, Barbara Mantovani, Daniela Di Sanzo, Vincenzo Gentili, Massimo Carletti, Massimo Aiuti, Andrea Gallo, Piero Giuseppe Meliante, Salvatore Martellucci, Oliviero Riggio, Vincenzo Cardinale, Lorenzo Ridola, Maria Consiglia Bragazzi, Stefania Gioia, Emiliano Valenzi, Camilla Graziosi, Niccolò Bina, Martina Fasolo, Silvano Ricci, Maria Teresa Gioacchini, Antonella Lucci, Luisella Corso, Daniela Tornese, Parni Nijhawan, Francesco Equitani, Carmine Cosentino, Marcello Palladino, Frida Leonetti, Gaetano Leto, Camillo Gnessi, Giuseppe Campagna, Roberto Cesareo, Francesca Marrocco, Giuseppe Straface, Alessandra Mecozzi, Lidia Cerbo, Valentina Isgrò, Sergio Parrocchia, Giuseppe Visconti, Giorgio Casati, Carlo Calzetti, Alarico Ariani, Lorenzo Donghi, Nicola Duccio Salerno, Evelina Tacconelli, Marco Bertoldi, Paolo Cattaneo, Lorenza Lambertenghi, Leonardo Motta, Luca Omega, Giovanni Albano, Roberto Parrella, Fiorentino Fraganza, Luigi Atripaldi, Vincenzo Montesarchio, Francesco Scarano, Annunziata De Rosa, Amalia Buglione, Sabrina Lavoretano, Gianfranco Gaglione, Mario De Marco, Vincenzo Sangiovanni, Francesco Maria Fusco, Rosaria Viglietti, Elio Manzillo, Carolina Rescigno, Raffaella Pisapia, Giulia Plamieri, Alberto Maraolo, Giosuè Calabria, Mario Catalano, Giuseppe Fiorentino, Anna Annunziata, Giorgio Polistina, Pasquale Imitazione, Mariano Mollica, Vincenzo Esposito, Maurizio D’Abraccio, Rodolfo Punzi, Vincenzo Bianco, Costanza Sbreglia, Rosa Fontana Del Vecchio, Alessandro Bordonali, Antonina Franco, Carlo Salvarani, Marco Massari, Giovanni Dolci, Pierpaolo Salsi, Giuseppe Virzì, Calderone Ornella, Alfredo Molteni, Silvia Gennarini, Umberto Gnudi, Maria Anastasia Ricci, Giancarlo Titolo, Giulio Mensi, Pietro Vuotto, Beatrice Gasperini, Mauro Mancini, Zeno Pasquini, Paolo Spanu, Stefano Clementi, Simona Pierini, Daniela Bokor, Daniela Gori, Morena Ciofetti, Marina Caimi, Laura Bettazzi, Elisabetta Allevi, Silvia Furiani, Chiara Capitanio, Bernardino Mastropasqua, Claudio Fara, Grazia Pulitanò, Jun Sebastian Matsuno, Francesca Della Porta, Viola Dolfini, Nebiat Balei Beyene, Michela Bezzi, Mauro Novali, Pierluigi Viale, Sara Tedeschi, Renato Pascale, Raffaele Bruno, Alessandro Di Filippo, Michele Sachs, Tiberio Oggionni, Michele Di Stefano, Caterina Mengoli, Cesarina Facchini, De Nardo Daniele, Gabriele Frausini, Luciano Mucci, Silvia Tedesco, Rita Girolimetti, Elena Manfredini, Anna Maria Di Carlo, Emma Espinosa, Donatella Dennetta, Andrea Ticinesi, Tiziana Meschi, Antonio Nouvenne, Norbiato Claudio, Francesco Vitale, Marta Saracco, Mauro Codeluppi, Elisa Fronti, Patrizia Ferrante, Giorgio Amadio Nespola, Daniela Francisci, Andrea Tosti, Cristiano Matteo Carbonelli, Antonio Greco, Maria Giulia Tinti, Roberto Stellini, Camilla Appiani, Piera Reghenzi, Venerino Poletti, Claudia Ravaglia, Danilo Tacconi, Costanza Malcontenti, Pier Paolo Sainaghi, Raffaella Landi, Veronica Vassia, Eleonora Rizzi, Mattia Bellan, Antonella Rossati, Luigi Castello, Claudio Maria Mastroianni, Gianluca Russo, Toffoletto Fabio, Francesco Saverio Serino, Lucio Brollo, Elena Momesso, Maria Luisa Turati, Antonella D’arminio Monforte, Giulia Marchetti, Fabrizio Boni, Elisabetta Teopompi, Chiara Trenti, Luca Boracchia, Enrica Minelli, Matteo Fontana, Giulia Ghidoni, Anaflorina Matei, Andrea Caruso, Giuseppe Arcoleo, Gaetana Camarda, Filippo Catalano, Mario Spatafora, Donato Bettega, Massimo Andreoni, Elisabetta Teti, Loredana Sarmati, Andrea Di Lorenzo, Mariagrazia Celeste, Fabio Baratto, Jacopo Monticelli, Pietro Criveller, Antonini Andrea, Anselmo, Riccio, Maurizio Castellano, Carlo Cappelli, Federica Corvini, Barbara Zanini, Massimo Crippa, Maurizio Ronconi, Raffaella Costa, Silvia Casella, Loretta Brentana, Livio Bernardi, Andrea Frascati, Sandro Panese, Fabio Presotto, Lucio Michieletto, Cristina Bernardi, Maurizio Fusar, Vanni Agnoletti, Martina Farina, Russo, Federico Lavorini, Roberta Ginanni, Fabrizio Palmieri, Silvia Mosti, Angelo Amaglio, Alessandra Cattaneo, Silvia Cirri, Andrea Montisci, Chiara Gallazzi, Daniele Cosseta, Barabara Baronio, Lorenzo Rampa, Paolo Maggi, Vincenzo Messina, Emanuele Alberto Negri, Maria Cecilia Sabatti, Michele Palumbo, Antonino Mazzone, Paola Faggioli, Linda Bussini, Giacomo Fornaro, Francesca Volpato, Daniele Imperiale, Emilpaolo Manno, Enrico Ferreri, Domenico Martelli, Andrea Verhovez, Silvia Giorgis, Luciana Faccio, Rachele Delli Quadri, Cristina Negro, Marcella Converso, Francesca Bosco, Paolo Prandini, Silvia Cocchi, Vinicio Manfrin, Veronica Del Punta, Giovanni Mazzola, Giuseppe Sportato, Micaela Romagnoli, Francesco Cristini, Francesca Facondini, Tiziana Perin, Andrea Boschi, Cristina Mussini, Marianna Meschiari, Giovanni Guaraldi, Sara Modica, Sara Moneta, Daniela Boccalatte, Clara Ricci, Valentina Marchetti, Silvia Amadasi, Gabriele Ebbreo, Michael Dalè, Paolo Tura, Damiano Rizzoni, Gianluca Edoardo Mario Boari, Silvia Bonetti, Enrico Marini, Italiani Daniele, Paolo Antonio Grossi, Nichola Walter Delfrate, Othmar Bernhart, Gilbert Spizzo, Klaus Mahlknecht, Thomas Volkl, Massimo Antonio Di Pietro, Michele Trezzi, Cecilia Monacci, Adriano Peris, Manuela Bonizzoli, Luigi Cavanna, Carlo Moroni, Elisa Maria Stroppa, Alessandra Manini, Maria Cristina Savio, Francesca Gatti, Clara Bartolaminelli, Nicola Petrosillo, Davide R. Donno, Fabrizio Taglietti, Simone Topino, Pierangelo Chinello, Vincenzo Galati, Gianpiero D’offizi, Chiara Taibi, Barbara Cimolato, Federico Moroni, Nicolas Palagano, Lorenzo Pelagatti, Seravalle Cristiana, Giancarlo Landini, Maria Amitrano, Mariangela Raimondo, Sara Mangiacapra, Annamaria Romano, Mariangela Atteno, Katia Casinelli, Ilaria Uccella, Sergio Harari, Antonella Caminati, Filippo Lipani, Giovanni Di Perri, Andrea Calcagno, Guido Calleri, Chiara Montrucchio, Anna Maria Caputo, Susanna Cozzio, Livia Delle Donne, Matteo Bassetti, Mikulska Malgorzata, Laura Ambra Nicolini, Chiara Russo, Chiara Sepulcri, Sabrina Beltramini, Federica Mina, Massimo Puoti, Anna Gandino, Thomas Langer, Federico D’amico, Marialma Berlendis, Chiara Rocchetti, Francesca Cettolo, Frausini Gabriele, Pietro Bocchi, Giorgio Cioni, Cinzia Cappi, Silvia Corcione, Francesco Giuseppe De Rosa, Silvia Scabini, Francesca Canta, Simone Mornese Pinna, Anna Pensa, Pierluigi Blanc, Lorenzo Roberto Suardi, Carlo Pallotto, Monica Rocco, Maria Teresa Cirasa, Michele Spinicci, Jessica Mencarini, Lorenzo Zammarchi, Cenderello Giovanni, Katiuscia Sciolè, Flavio Bassi, Michele Bianchi, Sillia Frigerio, Sandra Spaziani, Antonia Nucera, Giuliano Rizzardini, Maria Vittoria Cossu, Marco Antivalle, Giuseppe Carpinteri, Sebastiano Macheda, Demetrio Labate, Maurizio Bottiroli, Anacleto Romano, Elke Maria Erne, Zocchetti Cristina, Valeria Di Biase, Fabio Malberti, Giovanni Montani, Paolo Poisa, Daniela Bettini, Roberto Cauda, Arturo Ciccullo, Niccolò Riccardi, Andrea Angheben, Mauro Turrini, Raffaella Clerici, Angelo Gardellini, Luigi Liparulo, Tizana Rossini, Claudio Ucciferri, Francesco Cipollone, Jacopo Vecchiet, Andrea Nico, Lorenzo Marra, Armando Leone, Antonia Sdanganelli, Giuseppe Antonio Palmiotti, Giancarlo D’Alagni, Teresa Antonia Santantonio, Sergio Lo Caputo, Irene Bottalico, Antonio Ponticiello, Felicia Di Perna, Enrico Bernardi, Angela Beltrame, Stefania Bravi, Marco David, Paola Bernardi, Dario Galante, Maria Cristina Uccelli, Katiela Prestini, Monica Drera, Enea Zini, Alessio Peregrinelli, Laura Blanzuoli, Valentina Benedetti, Rovberta Calvi, Nadia Scaglione, Gabriella Nallino, Maurizio Bonazzi, Tiziano Crespi, Tiziana Masolin, Angelo Regazzetti, Maria Chiara Cerri, Elena Maffezzini, Manuela Piazza, Claudia Papetti, Claudia De Filippi, Elena Roveda, Giuseppe Cipolla, Mariano Scozzafava, Monica Crepaldi, Sonia Henchi, Nicolò Vanoni, Alice Repossi, Monia Vezzoli, Eva Scorletti, Orietta Perugini, Simone Marino Pasini, Veronica Pacetti, Luisella Ferrari, Giovanna Attilia de Paduanis, Sara del Duca, Francesca dell’Ara, Alessandra Brocchieri, Guja Minoja, Enrico Storti, Loredana Pitagora, Isabella Costa, Fanny Delfanti, Matteo Orlandi, Ruggero Ruggeri, Lorenzoa Ruggieri, Sergio Livigni, Daniela Silengo, Walter Ageno, Luciano Pedrini, Stefania Artiol, Laura Morbidoni, Giuseppe De Donno, Viviana Ravagnani, Francesco Inglese, Pier Giorgio Scotton, Paolo Costantini, Maurizio Delucchi, Enrico Clini, Andrea Ansuini, Baiocchi Marco, Lain Giuseppe, Brianti Vincenzo, Gianni Rastelli, Andrea Doria, Andrea Vianello, Anna Maria Cattelan, Sara Bindoli, Mara Felicietti, Ciro Canetta, Alessandro Scartabellati, Silvia Accordino, Maurizio Ferrara, Livio Cocco, Fernanda Cirillo, Erminio Pace, Monica De Caro, Marielisa Alberico, Giovanni Benigni, Terenzio Damiano, Pierluigi Fusco, Angela Iuorio, Giacomo Torretta, Milena Racagni, Stefano Muttini, Girolamo Sala, Paolo Ghiringhelli, Fernando Chiumiento, Laura Baccari, Boracchia Luca, Federica Bocchi, Francesco Benatti, Jacopo Catellani, Marina Coppola, Alberto Papi, Enrico Bosco, Mnuela Bonizzoli, Chiara Lazzeri, Nencioni Cesira, Camilla Puttini, Tiziana Carli, Leonardo Croci, Marta Corridi, Massimo Arlotti, Giulio Guerrini, Luisanna Cola, Michela Romanelli, Marina Bonifazi, Stefano Gasparini, Federico Mei, Elisabetta Cerutti, Donato Lacedonia, Armando Santoro, Giacomo Maria Guidelli, Stefano Greco, Andrea Castellan, Gessica Infantino, Laura Camici, Francesca Covani Frigieri, Vittorio Pavoni, Lucia Migliori, Barbara Rossetti, Francesca Montagnini, Immacolata Mauro, Elvira Genovese, Antonio Capuozzo, Leonarda Vitiello, Emanuele Sirignano, Paolo Gnesin, Giuseppe Servillo, Alfredo Marinelli, Daniela Pasero, Sergio Babudieri, Giordano Madeddu, Andrea De Vito, Lorenzo Casadio, Melania Ranghitta, Rodolfo Passalacqua, Fioravanti Antonio, Ivan Gentile, Antonio Riccardo Buonomo, Riccardo Scotto, Emanuela Zappulo, Giuseppina Dell’Aquila, Angel Bianchetti, Fabio Guerini, Alfredo Vallone, Peppino Oppedisano, Luigi Pusterla, Omar Giglio, Grazia Russo, Enrico Sartori, Cristina Zanardini, Pietro Gatti, Valiani Vincenzo, Stefania Piconi, Chiara Molteni, Giuseppina Dognini, Franco Cosimo, Luigi Guarneri, Fabrizio Pulvirenti, Vincenzo Mondino, Gabriella Traballi, Enrico Iemoli, Antoenlla Grisolia, Riccardo Giorgi, Gabriella Nucera, Valentina Raffaelli, Pietro Marino, Enrica Negro, Lisa Serati, Tamanini Silvia, Carmelo Iacobello, Giuseppe Strano, Lucio Boglione, Alberto Catania, Paola Gipponi, Luca Di Cato, Anna Panaccione, Giovanni Vitale, Ilaria Alice Crippa, Matteo Giacomini, Adriano Basile, Bellone Andrea, Paolo Tundo, Stefano Buzzigoli, Gerardo Palmiero, Andrea Magnaca, Matteo Silva, Massimo Ricci, Stefano Crespi, Bernadetta Pasquino, Guglielmo Consales, Damiano Bragantini, Franco Mastroianni, Giulia Righetti, Antonio Scarafino, Michele Bitetto, Fabio Franzetti, Sandro Piga, Vito Delmonte, Sergio Carbonara, Ruggero Losappio, Christian Dejaco, Claudio Mastroianni, Valerio Del Bono, Fabio Gilioli, Daniela Barzan, Silvia De Struppi, Antonio Carlotto, Maria Licia Guadagnin, Massimo Girardis, Elisabetta Bertellini, Francesco Dentali, Giancarlo Foresta, Alberto Baratta, Rosangela Viviani, Antonio Maria Agrati, Giovanni Battista Perego, Arturo Montineri, Rosa Manuele, Salvatore Bonfante, Donatella Aquilini, Alessandra Prozzo, Donato Santopuoli, Zaira Di Rosa, Armando Alborghetti, Paolo Peci, Nikoloz Bakhtadze, Chiara Stefania Pandini, Giovanni Casati, Najat Ashofarir, Giampaolo Casella, Walter Spagnolli, Silvana Urru, Ivan Marchesoni, Giulia Caminiti, Elena Argilloni, Elisabetta Danieli, Gianluca Ghirardi, Chiara Maria Antonioli, Alessio Lipari, Paola Zavarise, Francesco Kokaly, Enrico Polati, Leonardo Gottin, Paolo Lucernoni, Fabio De Conti, Elisabetta Marcon, Emanuele Pontali, Elisabetta Blasi Vacca, Carolina Saffioti, Alessia Zunino, Erik Roman Pognuz, Giorgio Berlot, Martino Saltori, Andrea Tedesco, Carlo Agostini, Maria Antonietta Di Rosolini, Francesco Marino, Guido Bellinzona, Walter Grassi, Marco Di Carlo, Guglielmo Scimonello, Sandra Nonini, Michele Mondino, Lorenzo Filippo Mantovani, Elena Tenti, Concetta Maria Gabriella Tropea, Daniela Emanuela Di Stefano, Paolo Guelfi, Lorenzo Dagna, Gianfranco Morgana, Lidia Montemurro, Domenico Girelli, Ernesto Crisafulli, Alessio Maroccia, Alessandra Maria Cemuschi, Mara Bernasconi, Ugo Zummo, Valentina Barbato, Simona Bevilacqua, Gaetano Buonfanti, Giuliana Canzanella, Giovanni De Matteis, Manuela Florio, Marilena Martino, Maria Teresa Ribecco, Fiorella Romano, Alfonso Savio, Lucia Sparavigna, Marcello Curvietto, Michele Citarella, Valeria Nava, Paola Maggioni, Marta Magni, Chiara Iommelli, Antonella Bianco, Romina Corsini, Linda Valli, Maria Paola Ruggieri, Tiziana Melica, Alessandra Ferrari, Daniela Cicognini, Mariangela Delliponti, Andrea Zuccarini, Silvia Ciani, Davide Raffaeli, Luca Donati, Stefania Cannizzo, Stefania Lui, Luca Santini, Enrica Roncaglia, Pasquale Mighali, Frederik Eisendle, Giulia Cerino, Chiara Citterio, Camilla Di Nunzio, Annalisa Mancini, Silvia Lamonica, Silvia Resimini, Giovanni Sarteschi, Chiara Pavei, Nicholas Battistini, Ospedale Erika Gazzola, Marco Miceli, Silvia Pontiggia, Veronica Lonati, Giusy Giannandrea, Claudio Sortino, Serena Ravani, Cristiano Uggeri, Genny Jocollé, Cristina Baré, Irene Baroni, Daniele De Candia, Barbara Fiorini, Katiuscia Chierico, Francesca Romeo, Roberta Bottega, Laura Boccasile, Annamaria Corsaro, Claudia Spadoni, Silvia Chiari, Giovanna Ercolino, Vanessa Dell’uomo, Sabrina Viri, Milena Minato, Lisa Gazzola, Balan Dorina, Davide Gianelli, Sonia Maspero, Maddalena Farinazzo, Paola Zanini, Antonella Sangiovanni, Antonia Del Giudice, Maria Margherita Dragonetti, Susanna Bordignon, Andrea Marco Machiavelli, Giulia Chiodelli, Micaela Spatarella, Davide Zenoni, Flavio Niccolò Beretta, Giuseppina Santilli, Rita Badagliacca, Manuela Angileri, Luciana Giannelli, Annalisa Campomori, Patrizia Maimone, Andrea Fadda, Sonia Faoro, Alessia Pisterna, Bruno Cacopardo, Andrea Marino, Alessio Pampaloni, Benedetto Maurizio Celesia, Gilda Cinnella, Daniela Labella, Rosa Roberta Caporusso, Maria Danzi, Marta Fiscon, Marina Malena, Doris Fendt, Stefano Nardi, Paolo Stobbione, Maria Laura Savi, Andrea De Monte, Alberto Scala, Nicola Lucio Liberato, Sauro Luchi, Antonella Vincenti, Luca Cabrini, Giovanni Pinelli, Lucio Brugioni, Domenico Potenza, Fabio Giuliano Numis, Giovanni Porta, Maria D’amico, Bianca Iengo, Gioacchino Angarano, Annalisa Saracino, Livio Blasi, Pasquale De Negri, Stefano Angelici, Antonella Farina, Giuseppe Pio Martino, Giuseppina Bitti, Alberto Tedeschi, Simona De Ponti, Adriana Agostinone, Giustino Parruti, Augusta Consorte, Antonella Frattari, Amelia Filippelli, Pasquale Pagliano, Alfonso Masullo, Carmine Sellitto, Massimo Reta, Nicolò Rossi, Luigi Raumer, Silvia Andreassi, Paolo Brancaleoni, Antonina Carai, Anna Maria Salerno, Franco Marinangeli, Roberta Mariani, Antonello Ciccone, Carlo Meschini, Gianluca Santoboni, Claudio Angrisani, David Micarelli, Giovanna Tarquini, Vittorio Fregoni, Carlo Alberto Volta, Antonio Cherubini, Maria Simona Del Prete, Erika Ciarrochi, Francesca Tasca, Andrea Ballarin, Andrea Bianchin, Romeo Flocco, Vincenzo Cuzzone, Maurilio Carpinteri, Pietro Gallotti, Federica Torre, Pio Zannetti, Massimo Crapis, Sergio Venturini, Massimo Barattini, Gianluca Gori, Antonio Mastroianni, Giulio De Stefano, Michele Gilio, Giuseppe Rapisarda, Leonardo Gulisano, Maria Luisa Granata, Sebastiana Saglimbene, Maria Teresa Montalto, Ilaria Grasso, Silvana De Luca, Gaetano Magro, Florinda Messina, Bruno Scapino, Paolo Abrate, Chiara Francisco, Laura Pesce, Mauro Navarra, Michele Agosti, Silvia Pagani, Martina Piluso, Aristodemo Ricioppo, Silvia Tognella, Pierangelo Rovere, Marcello Vincenzi, Leonardo Ghirardi, Daniele Generali, Marco Ingrosso, Emilia Desiderio, Raffaele Molaro, Silvio Vitiello, Alessandro Mastroianni, Luca Lancione, Tonia Celeste Paone, Alessandro Meli, Stefano Mainardi, Valentina Rastellino, Antonia Ursillo, Paola di Grigoli, Elena Bovetto, Ivana Marina Stefanetto, Francesca Mazzola, Antonio Daniele, Claudia Bisio, Pietro Delnero, Giovanni Morando, Antonella Nava, Lemut Francesco, Fabio Fiammengo, Monica Regis, Dario Roccatello, Eugenio Sabato, Marco Maria Liccardi, Cristina Bretto, Lorenzo Lutri, Enzo Castenetto, Giuseppe Roberti, Maria Francesca Guidi, Francesco Bini, Maria Crisitna Zappa, Tiziana Trequattrini, Rosario Rivitti, Rossana Vigliarolo, Angela Succu, Marianna Lilli, Mattia Serao, Giuseppina Giogré, Annamaria Ruggieri, Kristoffer Flores, Giuseppe Vairo, Roberto Satira, Anna Lingua, Rosario Spina, Emanuele Nicastri, Gaetano Maffongelli, Filippo Barreca, Sabino Scollet, Federico Franchi, Camilla Fabbri, Pietro Minuz, Andrea Dalbeni, Paolo Zanatta, Domenico Gelormini, Anna Mandelli, Feliciano Galderisi, Elena Zoia, Maria Rita Marchi, Naile De Almeida Neves, Giorgio Carbone, Emilio Di Caterino, Anna Petrone, Carlo Andrea Usai, Francesco Bandiera, Roberto Monti, Alex Hofer, Giacomo Castiglione, Chiara Angeletti, Paolo Tarsia, Lorenzo Veronese, Paola Daniela Artoni, Dora Larussa, Roberto Fumagalli, Paolo Brioschi, Alessandro Cerutti, Paola Pasquino, Fiore Gilberto, Luca Cantadori, Gabriele Tomasoni, Lina Rachele Tomasoni, Nicola Coppola, Stefano Spolveri, Costanza Pollastri, Lorenzo Fico, Tiziana Principi, Silvia Pierantozzi, Costantino Fontana, Giuseppe Lubrano, Laura Martinelli, Stefano Bravi, Paolo Navalesi, Eugenio Serra, Enrico Cogi, Andrea Manzi, Ermenegildo Furino, Nicola Dasseni, Claudio Gentilini, Elisa Benatti, Andrea Pignatti, Giuseppe Aiello, Mario Milia, Maria Grazia Covesnon, Annalisa Brianti, Claudio Francesco, Blangetti Ilaria, Fiammetta Pagnozzi, Sabrina Mietta, Alberto Rossi, Lorenzo Maroni, Vittorio Borroni, Claudio Bellintani, Camilla Sgarabotto, Giada Bizzotto, Lucio Bucci, Giovanni Spagnuolo, Moreno Agostini, Federico Carlo Caria, Filippo Testa, Raffaele De Palma, Giuseppe Murdaca, Gabriele Zanolini, Nadia Sala, Erminio Righini, Roberto Pontremoli, Gianmarco Aondio, Ferdinando Riccardi, Maria Giovanna De Cristoforo, Fausto De Michele, Angelo Storti, Roberto Perra, Silvia Deidda, Caviglia Enrica, Federico Valastro, Matteo Giorgi Pierfranceschi, Fabio De Gennaro, Anna Laura Nardecchia, Mariateresa Castellini, Giovanni Buetto, Giovanbattista Ippoliti, Domenico Sicheri, Maria Grazia Bottoli, Blanca Martinez Lopez De Arroyabe, Alessandra Versaci, Casa Di Cura Villa Giada Pallotti, Marina Civita, Michele Grio, Nicola Liuzzi, Paola Molino, Mauro Pastorelli, Alberto Ricchiardi, Ferdinando Varbella, Angela Daniela Zeme, Cinzia Sighieri, Grazia Portale, Alessandro Olivetti, Carlo Pagnoni, Greta Moschini, Sabrina Boni, Alberto Guerra, Roberta Scudellari, Sabrina Vella, Sandro Inchiostro, Ornella Piazza, Salvatore Guarino, Giorgio Aldegheri, Giovanna Napoli, Alessandro Morettini, Eleonora Caldini, Lorenzo Menicacci, Filippo Pieralli, Monica Torrini, Loredana Poggesi, Enrico Maria Visetti, Carmelo Mangano, Stefano Visconti, Pasquale Maietta, Elisa Banfi, Stefania Cartella, Bruna Venturi, Antonia Nuceri, Elena Chiesa, Enrico Pacentra, Gianluigi Panzolato, Michella Giannotti, Cristina Bianchi, Antonello Pietrangelo, Ombretta Para, Maria Serena Rutili, Roberto Russo, Maurizio Lanfranco, Elisa Scalabrino, Agostino Tafuri, Elisa Perfetti, Tosca Chiarello, Luca Cancanelli, Manuela Otero, Greta Pannella, Francesco Bellucci, Giovanna Ferrero, Carmen Vico, Maria Serafina Stillante, Giovanna D’Andrea, Filippo Amoroso, Antonio Arcidiacono, Anna Maria Bella, Agata Belsito, Ylenia Berté, Giulia Carubia, Maria Grazia Caruso, Orazio Casella, Francesco Chiereleson, Chiara Costa, Daniela De Franco, Giuseppe Germanà, Antonio Messina, Diana Musumeci, Concetta Noto, Marco Valenti, Carlo Sorrentino, Rosanna Panico, Giuseppe Schettino, Jolanda Piccoli, Antonio Pepe, Francesco De Rosa, Mario Ottaviano, Gerarda Marrazzo, Gianna Raponi, Stefania Diberardino, Simona Bausi, Alessandro Ferrari, Sara Francesca Marini, Elena Giubellino, Giorgio Innocenti, Gaspare Gugliemi, Daniela Maccari, Izabela Baciu

**Affiliations:** 1 Clinical Trial Unit, Istituto Nazionale Tumori, IRCCS, Fondazione G. Pascale, Napoli, Italy; 2 Melanoma, Cancer Immunotherapy and Development Therapeutics Unit, Istituto Nazionale Tumori, IRCCS, Fondazione G. Pascale, Napoli, Italy; 3 Rheumathology, Università degli Studi di Modena e Reggio Emilia and Azienda USL-IRCCS di Reggio Emilia, Modena, Italy; 4 Cotugno Hospital, AORN Ospedali dei Colli, Napoli, Italy; 5 Emilia Romagna Health Directorate, Bologna, Italy; 6 Center for Drug Research and Evaluation, Istituto Superiore di Sanità, Roma, Italy; 7 Infectious Diseases Unit, Hospital Health Direction, IRCCS - Policlinico San Donato, Milano Milano, Italy; 8 Infectious Diseases Unit - ASST Papa Giovanni XXIII, Bergamo, Italy; 9 Infectious Diseases Unit, ASST Monza and University Milano Bicocca, Milan, Italy; 10 University of Brescia and ASST Spedali Civili, Brescia, Italy; 11 Sapienza University of Rome, Santa Maria Goretti Hospital, Latina, Italy; 12 Infectious Diseases and Hepatology Unit AOU, Parma, Italy; 13 UOC Malattie Infettive e Tropicali, AOUI, Verona, Italy; 14 Anesthesia and Resuscitation Unit, Istituto Nazionale Tumori, IRCCS, Fondazione G. Pascale, Napoli, Italy; 15 Azienda USL-IRCCS di Reggio Emilia, Reggio Emilia, Italy; 16 Università degli Studi di Modena e Reggio Emilia, Modena, Italy; 17 Department of Mental Health and Preventive Medicine, Università degli Studi della Campania Luigi Vanvitelli, Caserta, Italy

**Keywords:** COVID-19, Pneumonia, Coronavirus, Tocilizumab, IL-6, Phase 2, Mortality, Safety

## Abstract

**Background:**

Tocilizumab blocks pro-inflammatory activity of interleukin-6 (IL-6), involved in pathogenesis of pneumonia the most frequent cause of death in COVID-19 patients.

**Methods:**

A multicenter, single-arm, hypothesis-driven trial was planned, according to a phase 2 design, to study the effect of tocilizumab on lethality rates at 14 and 30 days (co-primary endpoints, a priori expected rates being 20 and 35%, respectively). A further prospective cohort of patients, consecutively enrolled after the first cohort was accomplished, was used as a secondary validation dataset. The two cohorts were evaluated jointly in an exploratory multivariable logistic regression model to assess prognostic variables on survival.

**Results:**

In the primary intention-to-treat (ITT) phase 2 population, 180/301 (59.8%) subjects received tocilizumab, and 67 deaths were observed overall. Lethality rates were equal to 18.4% (97.5% CI: 13.6–24.0, *P* = 0.52) and 22.4% (97.5% CI: 17.2–28.3, *P* < 0.001) at 14 and 30 days, respectively. Lethality rates were lower in the validation dataset, that included 920 patients. No signal of specific drug toxicity was reported. In the exploratory multivariable logistic regression analysis, older age and lower PaO2/FiO2 ratio negatively affected survival, while the concurrent use of steroids was associated with greater survival. A statistically significant interaction was found between tocilizumab and respiratory support, suggesting that tocilizumab might be more effective in patients not requiring mechanical respiratory support at baseline.

**Conclusions:**

Tocilizumab reduced lethality rate at 30 days compared with null hypothesis, without significant toxicity. Possibly, this effect could be limited to patients not requiring mechanical respiratory support at baseline.

*Registration* EudraCT (2020-001110-38); clinicaltrials.gov (NCT04317092).

**Supplementary information:**

**Supplementary information** accompanies this paper at 10.1186/s12967-020-02573-9.

## Background

Pneumonia is the most frequent and serious complication of COVID-19, due to excessive host immune response causing an acute respiratory distress syndrome [1–5].

Interleukin 6 (IL-6) is a pro-inflammatory cytokine implicated in several rheumatic diseases and in the so-called cytokine release syndrome (CRS). Tocilizumab is a recombinant humanized monoclonal antibody, directed against the IL-6 receptor. It is indicated for treating severe rheumatoid arthritis, systemic juvenile idiopathic polyarthritis and severe cytokine release syndrome (CRS) induced by chimeric antigen receptor T-cells (CAR-T) [6, 7].

Chinese researchers treated 21 patients with severe or critical COVID-19 pneumonia with tocilizumab 400 mg iv with efficacy in terms of reduction of oxygen requirement (15/20), resolution of radiologic lung lesions (19/21), normalization of lymphocyte count (10/19), and reduction of C-reactive protein levels (16/19) [8]. These results prompted a randomised trial (tocilizumab vs control, ChiCTR2000029765).

On March 19th, 2020 during the ascending phase of the Italian breakout, we launched the TOCIVID-19 study, to describe the efficacy of tocilizumab while controlling the highly increasing off-label use of the drug.

## Methods

TOCIVID-19, an academic multicenter clinical trial, was promoted by the National Cancer Institute of Naples and was approved for all Italian centers by the National Ethical Committee at the Lazzaro Spallanzani Institute on March 18th, 2020; two amendments followed on March 24th, 2020 and April 28th, 2020 [9]. The study is coordinated through the web-based platform managed by the Clinical Trial Unit of the promoting center.

### Study design

330 patients were initially planned for the single-arm phase 2 study based on one-month lethality rate of 15% as null hypothesis, an alternative hypothesis for tocilizumab of 7.5% (i.e. halving the expected lethality rate), 99% power and 5% two-tailed alpha error. Taking into account about 20% of cases not eligible after registration 400 patients had to be enrolled. The initial calculation was based on March 10th daily report on Italian breakout, but data tumultuously accumulating between March 10th and April 15th clearly showed it was largely underestimated, and that adding an earlier outcome could be worthwhile. Thus, the April 24th amendment introduced 14-day lethality rate as co-primary endpoint, and the expected lethality rates (null-hypotheses) at 14 and 30 days were redefined at 2 and 35%, respectively, based on data received from the Italian National Institute of Health [10]. Nonetheless we decided to leave the planned sample size unchanged since it still allowed 99% and 95% power to recognize 10% absolute reduction at 14 and 30 days, respectively, with a significance level of 2.5% for each co-primary endpoint. It is worth emphasizing that any change in the protocol was introduced before extracting mortality data from the database, i.e. not being aware of the number and timing of recorded deaths.

### Patients

Patients hospitalized due to clinical/instrumental signs of pneumonia, and with real-time PCR diagnosed SARS-CoV-2 infection, were eligible for the phase 2 study if they had oxygen saturation at rest in ambient air ≤ 93% or required oxygen support or mechanical ventilation either non-invasive or invasive (intubated less than 24 h before registration). There was no limitation based on age and gender.

Patients were not eligible in case of known hypersensitivity to tocilizumab, known active infections or other clinical conditions that could not be treated or solved according to the judgment of the clinician and contraindicated tocilizumab, ALT/AST > 5 times the upper limit of the normality, neutrophils count < 500/mmc, platelets < 50.000/mmc, bowel diverticulitis or perforation.

Informed consent for participation in the study could be oral if a written consent was unfeasible. However, if patients lack capacity to consent due to disease severity, and an authorized representative was not immediately available, treatment could be administered by the treating physician on her/his own responsibility.

### Treatment

Tocilizumab was administered at the dose of 8 mg/kg up to a maximum of 800 mg per dose. Such dose is the same approved by FDA for the treatment of CRS following CAR-T therapy [6]. A second administration of tocilizumab (same dose) was allowed 12 h after the first one if respiratory function had not recovered, at discretion of the Investigator. Tocilizumab was supplied at no cost by Roche Italy. Due to the rapidly increasing request, a variable delay between the date of patient registration and drug availability at the clinical centers occurred. There was no contraindication for concomitant treatment of respiratory impairment; also, concomitant experimental antiviral treatment was allowed.

### Statistical analysis

Primary analysis was performed in the intention to treat population (ITT), defined as all patients enrolled; a secondary analysis was done in the modified ITT (mITT) population with patients who had received at least one dose of the study drug. All the subjects enrolled by uncooperative centers, i.e. centers providing information on baseline characteristics and treatment for less than 25% of their patients, were removed from any analyses. This amendment, in agreement with IDMC, was made blind to outcome data, i.e. before extracting mortality data.

Statistical analysis is detailed elsewhere [10]. Briefly, differences between groups of baseline characteristics, collected at the time of registration, are assessed for categorical variables using χ^2^ test and for continuous variables using Wilcoxon rank-sum test. Patients discharged to home or low-intensity care setting are considered alive at the end-date of the follow-up period of 30 days. Exact 97.5% Clopper-Pearson confidence intervals (CI) are calculated for the proportions of death at 14 and 30 days. Pre-specified null hypotheses at days 14 and 30 are tested by a two-sided binomial test with alpha level equal to 0.025. Efficacy outcomes (with exact 95% CI) are described in baseline subgroups defined by demographics and clinical variables and compared with exact χ^2^ test. Analyses were carried out using Stata version 14.0 (Stata Corp. College Station, TX, USA) and R version 3.6.1 (R Foundation for Statistical Computing, Vienna, Austria).

### Validation cohort

Since the number of patients planned in the single-arm phase 2 design was quickly achieved in less than 24 h, a second prospective cohort, involving eligible patients registered by participating centers in the five subsequent days, was added to the study to corroborate the main phase 2 findings. The analyses in this ‘validation’ cohort are to be considered secondary. The enrollment in the additional cohort was limited to five days because of the emerging drug shortage due to the huge request of drug by centers. The analyses performed in phase 2 were repeated in the validation cohort. For the sake of efficiency, the results of the validation cohort are reported side by side those of phase 2.

### Joint cohort for safety analysis

Analysis of safety was performed joining the two prospective cohorts and was limited to patients who received at least one dose of the study drug. Adverse events recorded from registration up to 30 days were graded according to CTCAE term (Version 5.0) and reported for each category and term as the worst grade suffered by patients through the whole period of observation after treatment administration.

### Exploratory multivariable analysis

An exploratory multivariable logistic regression model was also performed in the combined cohort to assess prognostic variables on survival, that involved treatment with tocilizumab and/or corticosteroids [11], age (≤ 60, 61–70, > 70), gender, type of respiratory support (oxygen, non-invasive mechanical ventilation [NIMV], invasive mechanical ventilation [IMV]), PaO2/FiO2 ratio (≤ 100, 101–200, > 200, missing/not evaluated), population (phase 2 or validation) and geographical area (Lombardia, Veneto, Emilia-Romagna, other Northern regions, Center, South and Islands) as covariates. To reduce immortal time bias, patients who received tocilizumab four or more days after registration were excluded from the analysis. The interaction effects between treatment and the other covariates were tested in turn one at a time by Wald test and retained in the final model only if significant. Difference in the lethality rate between treated and untreated patients was calculated within specific subgroups and 95% CI was calculated by means of Agresti and Caffo method [11]. Description of such differences must be considered as exploratory and hypothesis-generating only.

## Results

### Single-arm phase 2 cohort

**Fig. 1:**
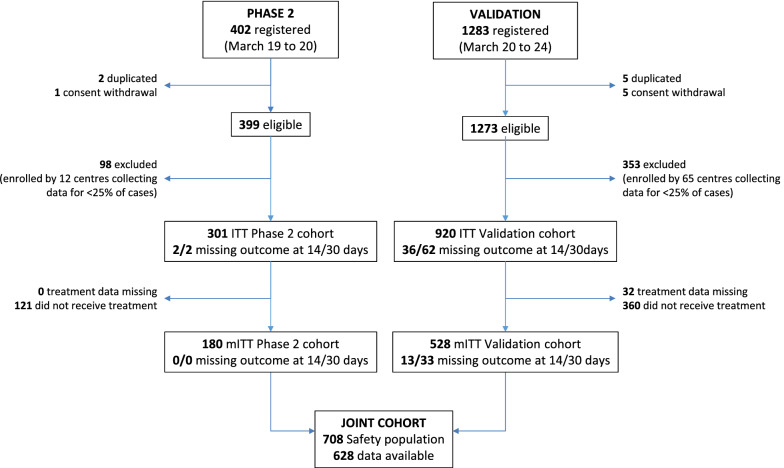
Study flow

From March 19th (at 14:00) to March 20th (at 12:45), 2020, 51 centers prospectively registered 402 patients for the phase 2 study (Fig. 1, left side), of which 2 cases were duplicated and one case withdrew consent. Ninety-eight patients enrolled by 12 uncooperative centers were removed from the analysis. Therefore, the phase 2 ITT population include 301 patients. Out of these, 21 were found ineligible a posteriori (12 intubated more than 24 h before registration, 7 registered after being already treated, 2 with both violations) but remained in the analysis. Geographical distribution and baseline characteristics of patients are summarized in Additional file 1: Figure S1 (top graphs), Table 1 (left side) and Additional file 1: Tables S1–3 (left side)**.**

**Table 1:** Baseline characteristics of patients in the ITT phase 2 and validation cohorts

	ITT Phase 2	ITT Validation
	N = 301	N = 920
Geographic area—no. (%)		
Lombardia	136 (45.2%)	346 (37.6%)
Veneto	65 (21.6%)	41 (4.5%)
Emilia Romagna	37 (12.3%)	142 (15.4%)
Other Northern regions	–	91 (9.9%)
Center	39 (13.0%)	186 (20.2%)
South and Islands	24 (8.0%)	114 (12.4%)
Age—no. (%)		
≤ 60	122 (40.5%)	375 (40.8%)
61–70	107 (35.5%)	263 (28.6%)
71 +	72 (23.9%)	282 (30.7%)
Female sex—no. (%)	59 (19.6%)	200 (21.7%)
Ethnic group—no. (%)		
Caucasian	271 (97.1%)	853 (97.7%)
Asiatic	3 (1.1%)	2 (0.2%)
Other	5 (1.8%)	18 (2.1%)
Unknown	22	47
Body mass index—no. (%)		
Underweight/normal (< 25)	75 (28.8%)	192 (26.9%)
Overweight/obese (25 +)	185 (71.2%)	521 (73.1%)
Unknown	41	207
Previous/actual smoker—No. (%)	51 (22.2%)	214 (29.2%)
Unknown	71	188
Antiflu 2019 vaccination—No. (%)	54 (25.0%)	121 (20.3%)
Unknown	85	325
Initial respiratory support—No. (%)		
Oxygen supplementation	146 (48.5%)	468 (50.9%)
NIMV	106 (35.2%)	359 (39.0%)
IMV	49 (16.3%)	93 (10.1%)
PaO2/FiO2 ratio—median (IQR)	136 (93,198)	154 (103,218)
PaO2/FiO2 ratio—No. (%)		
< 100	55 (32.4%)	129 (24.1%)
101–200	76 (44.7%)	244 (45.5%)
201–300	32 (18.8%)	116 (21.6%)
> 300	7 (4.1%)	47 (8.8%)
Missing or not tested	131	384
Comorbidities (mild or worse)—No. (%)		
Heart disease	62 (21.6%)	150 (18.1%)
Hypertension	147 (51.2%)	389 (47.0%)
Diabetes	34 (11.8%)	138 (16.7%)
Unknown	14	93
Concurrent treatment, no. (%)		
Antiretroviral	180 (63.1%)	576 (67.6%)
Hydroxy-chloroquine	207 (72.6%)	651 (76.4%)
Antibiotics	118 (41.4%)	443 (52.0%)
Steroids	62 (21.8%)	296 (34.7%)
LMW heparin	66 (23.2%)	175 (20.5%)
Unknown	16	68
C-reactive protein—median (IQR)	37.6 (14.7, 120.0)	36.3 (13.7, 137.0)
Missing or not tested	181	255

Due to lagged drug availability, treatment was given to 59.8% of patients. Median time from registration to treatment administration was 2 days; 23.3% of treated patients received tocilizumab four or more days after registration. The most frequent reason for not giving the drug (once available) was clinical improvement (Additional file 1: Table S4, left side). Patients who were younger, and those with worse respiratory function were preferentially treated; also, the geographic location of the center played a role (Table 2, left side).

**Table 2:** Distribution of baseline characteristics of patients collected at registration by treatment administration

	Phase 2			Validation		
	Treated (n = 180)	Not treated (n = 121)	*P*	Treated (n = 528)	Not treated (n = 360)	*P*
Geographic area—no. (%)			< 0.001			0.30
Lombardia	94 (52.2%)	42 (34.7%)		195 (36.9%)	140 (38.9%)	
Veneto	14 (7.8%)	51 (42.1%)		28 (5.3%)	12 (3.3%)	
Emilia Romagna	29 (16.1%)	8 (6.6%)		76 (14.4%)	65 (18.1%)	
Other Northern regions	–	–		51 (9.7%)	40 (11.1%)	
Center	23 (12.8%)	16 (13.2%)		107 (20.3%)	61 (16.9%)	
South and Islands	20 (11.1%)	4 (3.3%)		71 (13.4%)	42 (11.7%)	
Age—no. (%)			0.04			0.22
≤ 60	79 (43.9%)	43 (35.5%)		209 (39.6%)	156 (43.3%)	
61–70	67 (37.2%)	40 (33.1%)		148 (28.0%)	107 (29.7%)	
71 +	34 (18.9%)	38 (31.4%)		171 (32.4%)	97 (26.9%)	
Female sex—no. (%)	31 (17.2%)	28 (23.1%)	0.20	108 (20.5%)	85 (23.6%)	0.26
Ethnic group—no. (%)			0.42			0.1
Caucasian	170 (97.1%)	101 (97.1%)		494 (97.4%)	333 (97.9%)	
Asiatic	1 (0.6%)	2 (1.9%)		2 (0.4%)	0 (0.0%)	
Other	4 (2.3%)	1 (1.0%)		11 (2.2%)	7 (2.1%)	
Unknown	5	17		21	20	
Body Mass Index—no. (%)			0.06			0.74
Underweight/normal	40 (24.7%)	35 (35.7%)		112 (27.1%)	73 (26.0%)	
Overweight/Obese	122 (75.3%)	63 (64.3%)		301 (72.9%)	208 (74.0%)	
Unknown	18	23		115	79	
Previous/actual smoker—no. (%)	33 (22.4%)	18 (21.7%)	0.89	130 (30.2%)	79 (27.9%)	0.52
Unknown	33	38		97	77	
Antiflu 2019 vaccination—no. (%)	31 (21.5%)	23 (31.9%)	0.10	75 (21.8%)	44 (18.5%)	0.33
Unknown	36	49		184	122	
Initial respiratory support– no. (%)			0.003			< 0.001
Oxygen supplement	73 (40.6%)	73 (60.3%)		223 (42.2%)	223 (61.9%)	
NIMV	74 (41.1%)	32 (26.4%)		238 (45.1%)	112 (31.1%)	
IMV	33 (18.3%)	16 (13.2%)		67 (12.7%)	25 (6.9%)	
PaO2/FiO2 ratio– no. (%)			0.08			< 0.001
≤ 100	36 (33.6%)	19 (30.2%)		91 (25.9%)	30 (18.3%)	
101–200	53 (49.5%)	23 (36.5%)		170 (48.4%)	66 (40.2%)	
201–300	14 (13.1%)	18 (28.6%)		68 (19.4%)	44 (26.8%)	
> 300	4 (3.7%)	3 (4.8%)		22 (6.3%)	24 (14.6%)	
Unknown	73	58		177	196	
Heart disease—no. (%)	31 (17.8%)	31 (27.4%)	0.053	99 (19.4%)	48 (15.6%)	0.17
Unknown	6	8		18	53	
Hypertension—no. (%)	92 (52.9%)	55 (48.7%)	0.49	242 (47.5%)	141 (45.9%)	0.67
Unknown	6	8		18	53	
Diabetes—no. (%)	23 (13.2%)	11 (9.7%)	0.37	84 (16.5%)	51 (16.6%)	0.96
Unknown	6	8		18	53	
Anti-retroviral—no. (%)	112 (65.1%)	113 (60.2%)	0.40	342 (66.4%)	224 (69.4%)	0.38
Unknown	8	8		13	37	
Hydroxy-chloroquine—no. (%)	130 (75.6%)	77 (68.1%)	0.17	395 (76.7%)	244 (75.5%)	0.70
Unknown	8	8		13	37	
Antibiotics—no. (%)	84 (48.8%)	34 (30.1%)	0.002	274 (53.2%)	163 (50.5%)	0.44
Unknown	8	8		13	37	
Steroids—no. (%)	41 (23.9%)	21 (18.6)	0.29	176 (34.2%)	115 (35.6%)	0.67
Unknown	8	8		13	37	
LMW heparin—no. (%)	45	221	0.14	116 (22.5%)	57 (17.7%)	0.09
Unknown	8	8		13	37	
C-reactive protein—median (IQR)	30 (13–116)	73 (17–122)	0.06	31 (14–132)	57 (14–144)	0.38
Unknown	34	29		102	128	

**Fig. 2:**
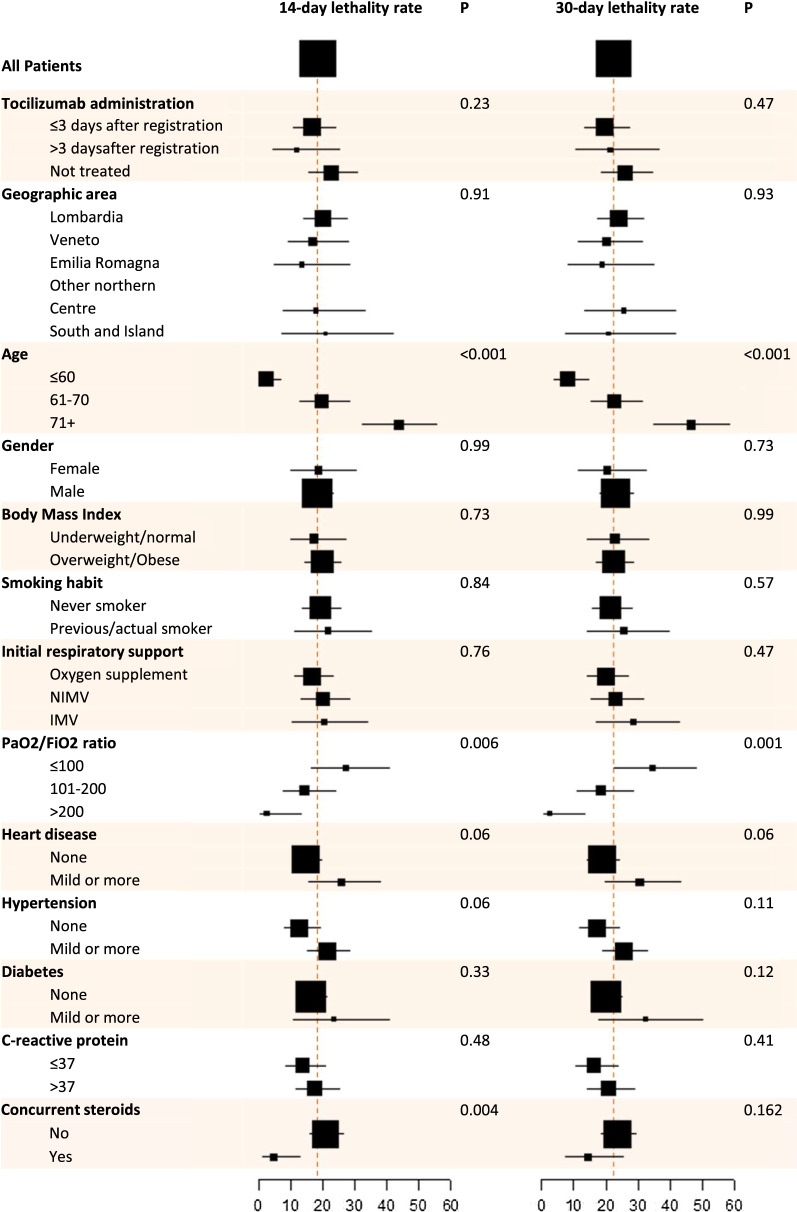
Estimated lethality rates at 14 and 30 days by baseline characteristics of patients in the phase 2 ITT population. Red dash lines represent lethality rates under null hypotheses

Overall, 67 (22.3%) deaths were reported in the ITT phase 2 cohort. Lethality rate was 18.4% (97.5% CI: 13.6–24.0) at 14 days and 22.4% (97.5% CI: 17.2–28.3) at 30 days. The null hypothesis was rejected at 30 days but not at 14 days (*P* < 0.001and *P* = 0.52, respectively). At both time points, lethality rates were lower in the mITT population (15.6% and 20.0%—Table 3, left side). Due to typical immortal time bias, lethality rates at 14 days were lower for patients receiving treatment four or more days after registration. Risk of death was significantly higher in patients older and with worse PaO2/FiO2 ratio; in addition, lethality rates were lower for patients receiving concurrent corticosteroids, particularly at 14 days where the difference was statistically significant (Fig. 2 and Additional file 1: Table S5, left side).

**Table 3:** Efficacy analysis

	Phase 2	Validation
14 days intention-to-treat		
No. of events/no. of patients at risk	55/299	101/884
Lethality rate, % (97.5% CI)	18.4% (13.6–24.0)	11.4% (9.1–14.0)
P value (P0 = 20%)	0.52	< 0.001
14 days modified intention-to-treat		
No. of events/no. of patients at risk	28/180	56/515
Lethality rate, % (95% CI)	15.6% (10.6–21.7)	10.9% (8.3–13.9)
30 days intention-to-treat		
No. of events/No. of patients at risk	67/299	158/858
Lethality rate, % (97.5% CI)	22.4% (17.2–28.3)	18.4% (15.5–21.6)
*P* value (P0 = 35%)	< 0.001	< 0.001
Median time of death, days (IQR)	8 (4–14)	11 (4–18)
30 days modified intention-to-treat		
No. of events/no. of patients at risk	36/180	99/495
Lethality rate, % (95% CI)	20.0% (14.4–26.6)	20.0% (16.6–23.8)

### Single-arm validation cohort

The validation cohort included 1273 patients enrolled by 211 centers from March 20th to March 24th, 2020 (Fig. 1, right side). Three hundred fifty-three patients enrolled from 65 uncooperative centers were removed, and 920 patients represented the ITT population. Baseline characteristics, shown in tables and figures side by side those of phase 2 patients, were more favorable in the validation than in the phase 2 cohort. Treatment compliance was similar (Additional file 1: Table S4, right side). Also in the validation cohort, available treatment was preferentially given to patients with worse respiratory function (Table 2, right side). Overall, 158 (17.2%) deaths were reported in the ITT validation cohort. Probability of death was lower in the validation than in the phase 2 cohort, particularly among untreated patients (Additional file 1: Figure S2). In the validation cohort, lethality rates were consistently lower than the predefined null hypothesis both at 14 and 30 days in the ITT (11.4 and 18.4%) and mITT (10.9% and 20.0%) populations (Table 3, right side). Subgroup analysis of lethality rates produced results similar to those seen in phase 2 (Additional file 1: Figure S3 and Table S5, right side).

### Safety analysis

Safety analysis was done in 628/708 patients of the combined cohort who had received at least one dose of tocilizumab (Additional file 1: Table S6). At least one adverse event was reported in 40.8% of patients. Of note, 68 deaths (10.8%) were categorized within adverse events scale. Causality between such deaths and treatment was described as possible only in one of the 35 cases of respiratory failure. All the other fatal adverse events were reported as unlikely or not related to treatment administration. Seven out of 8 fatal infections were specified as COVID pneumonia. Adverse events that may represent specific side effects of tocilizumab are allergic reactions [3 cases] and ALT or AST increase (reported in 10.5 and 9.1%, respectively) that was severe (grade 3 or 4) in around 3% of cases.

### Hypothesis-generating multivariable analysis

Results of the exploratory multivariable logistic regression analysis in the combined cohort are reported in Additional file 1: Table S7. Age and respiratory function measured by PaO2/FiO2 ratio were independently significant prognostic factors; the use of corticosteroids was associated with a lower OR of death both at 14 (OR 0.36, 95% CI: 0.21–0.62) and at 30 days (OR 0.62, 95% CI: 0.40–0.95). No significant interaction was found between the effect of tocilizumab and age, gender, PaO2/FiO2 ratio, geographic location and phase 2 vs validation cohorts; also, no interaction was found between the effect of tocilizumab and the use of corticosteroids. A significant interaction was found between treatment and required respiratory support, interaction test p-values being equal to 0.03 and 0.08 at 14 and 30 days, respectively. Specifically, treatment effect on lethality rates was larger among patients not requiring mechanical respiratory support within 24 h from registration with a OR equal to 0.37 (95% CI: 0.18–0.74) and 0.50 (95% CI: 0.27–0.92) and absolute reductions equal to 7.7 and 6.2%, at 14 and 30 days, respectively (Additional file 1: Figure S4).

## Discussion

The primary analysis of the single-arm phase 2 TOCIVID-19 cohort suggests that tocilizumab may reduce lethality at 30 days, although its impact at 14 days seems less relevant. The adverse event profile is consistent with other reports and did not generate clinically relevant warnings, possibly because of the severity of clinical symptoms related to the underlying pathologic condition. [12, 13] Interestingly, the exploratory multivariable analysis showed that the possible effect of tocilizumab might be greater among patients not requiring mechanical ventilation and might be independent of the effect of corticosteroids, that were associated with lower lethality rates, consistently with preliminary findings of the Recovery trial. [14] Further, we did not find an interaction between the effect of tocilizumab and the concurrent administration of corticosteroids, consistent with another recent report. [15].

In the light of the large percentage of untreated subjects (40%) and the selection bias of treating patients with worse prognosis, these results support using tocilizumab while waiting for the publication of results of the phase 3 clinical trials. To our knowledge, six ongoing randomised trials are comparing tocilizumab vs placebo (ChiCTR2000029765, NCT04320615, NCT04381936, EudraCT 2020-001408-41, NCT04330638, NCT2020-001767-86) and another one is comparing immediate vs delayed tocilizumab (NCT04346355). However, some trials have problems in reaching the planned sample size, and most of the trials on medical treatment of COVID-19 are using non validated surrogate outcomes rather than mortality as primary end-point [16].

TOCIVID-19 is the largest completed prospective study on the effect of tocilizumab using mortality as primary end-point, among published or pre-published reports. Mostly, retrospective or observational data have been reported so far, not based on prospective hypothesis testing, with prevalently positive results [17–32].

However, our study has several limitations that deserve discussion for a better interpretation of findings. The main limitation is the single-arm study design, which prevents definitive conclusions [33]. We did that because, in our opinion, a randomised controlled trial was unfeasible in the middle of March 2020 in Italy. Indeed, there was a tremendous pressure to have the drug available, due to a widespread media diffusion of positive expectations and the increasing number of patients hospitalized for the disease, as confirmed by the massive registration of centers when the study began. Physicians’ equipoise was poor, and obtaining a proper informed consent to randomization from patients was extremely difficult, because of clinical burden. Finally, developing a placebo was impossible, and, within a non-blinded study, the risk of cross-over from the control to the experimental arm would have been high, reducing the validity of the randomised trial. Within this context, the problem of “learning while doing” was increased, and we thought that the single-arm design was the best trade-off between do-something and learn-something [34].

A critical issue of the single-arm design was the definition of the null hypotheses to be tested. We amended them following the evolving information from the National Institute of Health when we were blind to outcome data and in agreement with IDMC [10]. Yet, we cannot be sure that our assumptions are unbiased. A study with data on about 43.000 patients coming from three Italian regions, reports higher lethality at 14 days (22.0%) and lower at 30 days (27.6%) compared to TOCIVID-19 null hypotheses; assuming these estimates as a benchmark, our results would be still clinically significant at both 14 and 30 days [35].

Difference of survival experience between the two cohorts was unexpected. However, due to the exceptional setting in which the study was conducted, the validation cohort allowed the appreciation of the heterogeneity of the study population. Thus, combining cohorts in the multivariable evaluation seemed the most reasonable approach to explore prognostic factors while adjusting for the many confounding factors.

An operational problem of our study was the discrepancy between timing of drug availability (notwithstanding the commitment of the pharma company) and the extremely high request due to the rapid recruitment rate. Two contrasting biases followed in our study: the indication (selection) bias, when physicians opted for treating patients with worse prognosis, and the immortal time bias, when delay of treatment administration favored subjects surviving longer enough to receive the drug. As expected, the latter bias was particularly evident at 14-day analysis. To be conservative, we excluded from multivariable analyses all patients receiving the drug later than three days from registration, and adjusted for all available confounding factors, although some residual bias may still exist. Thus, findings of the multivariable analyses are to be considered hypothesis-generating only.

Last, we had many missing data, for several reasons: massive involvement and stress of physicians in emergency care; paucity or absence of data-managers; quarantine of paper charts; impracticality of peripheral monitoring; lack of training to the web platform; slow web connections for the study platform due to huge information loading volume. In agreement with IDMC, we reduced the problem by removing un-cooperative centers that provided baseline information for less than 25% of patients; however, we cannot be confident that the remaining missing data are at random.

TOCIVID-19 also has some strengths. As mentioned above, it is the first academic trial promoted in Italy, the largest in terms of centers and patients (being available for the whole Italian territory), assessing a hard endpoint like mortality in a hypothesis-driven design, while off label use of the drug was increasing. [36] In addition, the internal validation, allowed by a companion prospective cohort, contributed to critical interpretation of the results. Further analyses will focus on secondary outcomes (e.g. respiratory outcomes, predictive and prognostic factors, epidemiology insights) and on a larger number of patients.

## Conclusions

Although with limitations of a single-arm study, performed in an extremely challenging time and environment, the present study supports the use of tocilizumab, even when corticosteroids are used, while waiting for publication of phase 3 results.

## Supplementary information

**Additional file 1.** TOCIVID-19_Appendix.

## Data Availability

The datasets used and/or analysed during the current study are available from the corresponding author on reasonable request.
