# Supplementary material for: Tocilizumab for patients with COVID-19 pneumonia. The single-arm TOCIVID-19 prospective trial
Source: J Transl Med. 2020 Oct 21;18:405. doi: 10.1186/s12967-020-02573-9 (PMC7576974; doi:10.1186/s12967-020-02573-9)
Supplement: Supplementary file 1 — Additional file 1. TOCIVID-19_Appendix. [file 12967_2020_2573_MOESM1_ESM.docx]

**Tocilizumab for patients with COVID-19 pneumonia. The TOCIVID-19 prospective phase 2 trial**

**Appendix**

**Summary**

[eTable 1. Distribution of patients by participating centres in the ITT phase 2 and validation cohorts 2](#_Toc44590938)

[eFigure 1. Geographic distribution of centres (red) and patients (green) in the phase 2 (top) and validation (bottom) cohorts 3](#_Toc44590939)

[eTable 2. Distribution of comorbidities (mild grade or worse) in the ITT phase 2 and validation cohorts 4](#_Toc44590940)

[eTable 3. Distribution of concurrent treatments in the ITT phase 2 and validation cohorts 5](#_Toc44590941)

[eTable 4. Details of compliance 6](#_Toc44590942)

[eFigure 2. Probability of death in the phase 2 (blue) and validation cohorts (orange) in the ITT (top) , mITT (middle) and untreated (bottom) populations. 7](#_Toc44590943)

[eTable 5. Lethality rates according to subgroups in ITT phase 2 and validation 8](#_Toc44590944)

[eFigure 3. Estimated lethality rates at 14 and 30 days by baseline characteristics of patients in the validation ITT population. Red dash lines represent lethality rates under null hypotheses. 10](#_Toc44590945)

[eTable 6. Worst per patient adverse events reported after treatment with tocilizumab by CTCAE grade 10](#_Toc44590946)

[eTable 7. Multivariable logistic regression 13](#_Toc44590947)

[eFigure 4. Relative (Odds Ratio of death, panel A) and absolute (difference in lethality rate, panel B) measures of tocilizumab effect by required respiratory support at 14 and 30 days. 14](#_Toc44590948)

[List of participating centres and Co-Investigators 14](#_Toc44590949)

# **eTable 1. Distribution of patients by participating centres in the ITT phase 2 and validation cohorts**

|  | **Phase 2 ITT**  **N=301** | **Validation ITT**  **N=920** |
| --- | --- | --- |
| **Specialty of the participating centre**, no. (%) |  |  |
| Infectious Disease | 204 (67.8%) | 341 (37.1%) |
| Internal Medicine (including subspecialties) | 71 (23.6%) | 378 (41.1%) |
| Intensive/Subintensive Care | 22 (7.3%) | 116 (12.6%) |
| Emergency/other | 4 (1.3%) | 85 (9.2%) |
| **Macroregion of the participating centre**, no. (%) |  |  |
| North | 238 (79.1%) | 620 (67.4%) |
| Centre | 39 (13.0%) | 186 (20.2%) |
| South and Islands | 24 (8.0%) | 114 (12.4%) |
| **Region of the participating centre**, no. (%) |  |  |
| Lombardia (57 centres) | 136 (45.2%) | 346 (37.6%) |
| Emilia-Romagna (25 centres including S.Marino) | 37 (12.3%) | 142 (15.4%) |
| Veneto (16 centres) | 65 (21.6%) | 41 (4.5%) |
| Piemonte (10 centres) | 0 (0.0%) | 56 (6.1%) |
| Liguria (4 centres) | 0 (0.0%) | 19 (2.1%) |
| PA Trento (1 centre) | 0 (0.0%) | 4 (0.4%) |
| PA Bolzano (2 centres) | 0 (0.0%) | 9 (1.0%) |
| Friuli Venezia Giulia (1 centre) | 0 (0.0%) | 3 (0.3%) |
| Lazio (10 centres) | 8 (2.7%) | 88 (9.6%) |
| Toscana (15 centres) | 16 (5.3%) | 36 (3.9%) |
| Marche (9 centres) | 3 (1.0%) | 55 (6.0%) |
| Umbria (2 centres) | 12 (4.0%) | 7 (0.8%) |
| Sicilia (5 centres) | 1 (0.3%) | 43 (4.7%) |
| Campania (13 centres) | 15 (5.0%) | 35 (3.8%) |
| Puglia (9 centres) | 8 (2.7%) | 19 (2.1%) |
| Abruzzo (3 centres) | 0 (0.0%) | 12 (1.3%) |
| Sardegna (1 centre) | 0 (0.0%) | 1 (0.1%) |
| Calabria (2 centres) | 0 (0.0%) | 4 (0.4%) |

# **eFigure 1. Geographic distribution of centres (red) and patients (green) in the phase 2 (top) and validation (bottom) cohorts**


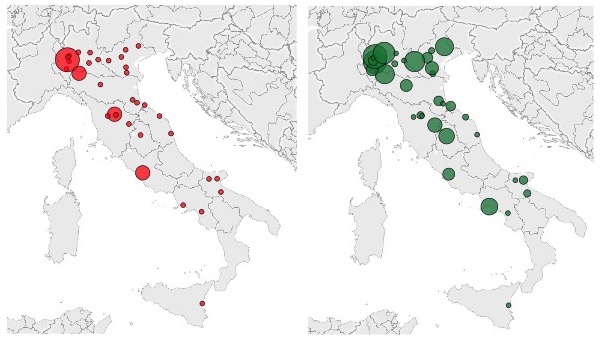


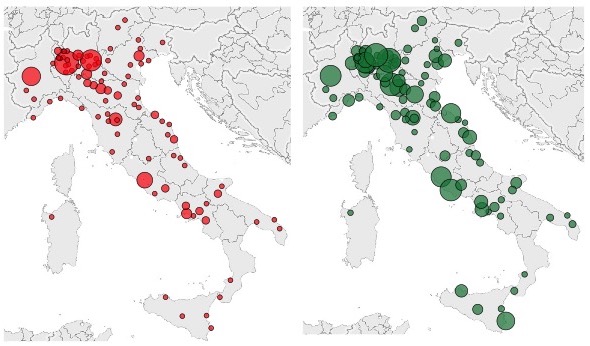


# **eTable 2. Distribution of comorbidities (mild grade or worse) in the ITT phase 2 and validation cohorts**

|  | **Phase 2 ITT**  **N=301** | **Validation ITT**  **N=920** |
| --- | --- | --- |
| ***Unknown*** | *14* | *93* |
| **Heart disease**, no. (%) | 62 (21.6%) | 150 (18.1%) |
| **Hypertension**, no. (%) | 147 (51.2%) | 389 (47.0%) |
| **Vascular disease**, no. (%) | 26 (9.1%) | 72 (8.7%) |
| **Respiratory disease**, no. (%) | 26 (9.1%) | 93 (11.2%) |
| **Ear Nose Throat**, no. (%) | 9 (3.1%) | 31 (3.7%) |
| **Upper GI tract disease**, no. (%) | 16 (5.6%) | 61 (7.4%) |
| **Lower GI tract disease**, no. (%) | 12 (4.2%) | 34 (4.1%) |
| **Liver disease**, no. (%) | 14 (4.9%) | 27 (3.3%) |
| **Kidney disease**, no. (%) | 20 (7.0%) | 44 (5.3%) |
| **Genito-urinary disease**, no. (%) | 16 (5.6%) | 82 (9.9%) |
| **Musculo-skeletal-skin disease**, no. (%) | 18 (6.3%) | 42 (5.1%) |
| **Nervous system disease**, no. (%) | 13 (4.5%) | 51 (6.2%) |
| **Diabetes**, no. (%) | 34 (11.8%) | 138 (16.7%) |
| **Psychiatric disorders**, no. (%) | 19 (6.6%) | 59 (7.1%) |
| **Cancer**, no. (%) |  |  |
| Present disease | 6 (3.8%) | 24 (4.6%) |
| Past disease | 14 (10.1%) | 54 (11.7%) |

# **eTable 3. Distribution of concurrent treatments in the ITT phase 2 and validation cohorts**

| **Class of drug or drug** | **Phase 2 ITT**  **N=301** | **Validation ITT**  **N=920** |
| --- | --- | --- |
| ***Unknown*** | *19* | *99* |
| **Antivirals**, no. (%) |  |  |
| Any antiviral | 179 (63.5%) | 549 (66.9%) |
| Lopinavir/Ritonavir | 148 (52.5%) | 409 (49.8%) |
| Remdesivir | 1 (0.4%) | 1 (0.1%) |
| Anti-HBV | 1 (0.4%) | 1 (0.1%) |
| Anti-HCV | 0 (0.0%) | 1 (0.1%) |
| Anti-flu | 0 (0.0%) | 4 (0.5%) |
| **(Hydroxy)chloroquine**, no. (%) | 205 (72.7%) | 627 (76.4%) |
| **Colchicine**, no. (%) | 0 (0.0%) | 3 (0.4%) |
| **Immune suppressor**, no. (%) | 1 (0.4%) | 7 (0.9%) |
| **Antibiotics**, no. (%) | 117 (41.5%) | 421 (51.3%) |
| Azythromicin | 51 (18.1%) | 169 (20.6%) |
| Ceftriaxone | 69 (24.5%) | 268 (32.6%) |
| Linezolid | 2 (0.7%) | 8 (1.0%) |
| **Steroids**, no. (%) | 61 (21.6%) | 277 (33.7%) |
| **LMW heparin**, no. (%) | 64 (22.7%) | 158 (19.2%) |
| **Anti-coagulants**, no. (%) | 16 (5.7%) | 35 (4.3%) |
| **Anti-aggregating**, no. (%) | 31 (11.0%) | 74 (9.0%) |
| **Anti-diabetics**, no. (%) | 23 (8.2%) | 55 (6.7%) |
| **Anti-hypertension**, no. (%) | 61 (21.6%) | 138 (16.8%) |
| ACE inhibitors | 11 (3.9%) | 18 (2.2%) |
| Sartane | 17 (6.0%) | 32 (3.9%) |
| NB. Missing cases are not considered in percentage calculation | | |

# **eTable 4. Details of compliance**

|  | **Phase 2 ITT**  **N=301** | **Validation ITT**  **N=920** |
| --- | --- | --- |
| ***Unknown*** | *0* | *32* |
| **Tocilizumab 1st dose**, no. (%) |  |  |
| Not administered | 121 (40.2%) | 360 (40.5%) |
| Death | 8 (6.6%) | 22 (6.1%) |
| Improvement | 37 (30.6%) | 162 (45.0%) |
| Worsening | 9 (7.4%) | 12 (3.3%) |
| Moved to other unit | 8 (6.6%) | 12 (3.3%) |
| Other | 5 (4.1%) | 19 (5.3%) |
| Lack of drug | 4 (3.3%) | 37 (10.3%) |
| Unknown | 50 (41.3%) | 96 (26.7%) |
| Administered | 180^a^ (59.8%) | 528^b^ (59.5%) |
| ≤3 days from registration | 138 (76.7%) | 404 (76.5%) |
| ≥4 days from registration | 42 (23.3%) | 124 (23.5%) |
| Median days (IQR) to administration | 2 (1-3) | 1 (0-3) |
| **Tocilizumab 2nd dose administered**, no. (%) |  |  |
| Not administered | 137 (45.5%) | 333 (37.5%) |
| Administered | 164 (54.5%) | 555 (62.5%) |
| ^a^ 7 cases subcutaneous; ^b^ 39 cases subcutaneous | | |

# **eFigure 2. Probability of death in the phase 2 (blue) and validation cohorts (orange) in the ITT (top) , mITT (middle) and untreated (bottom) populations.**


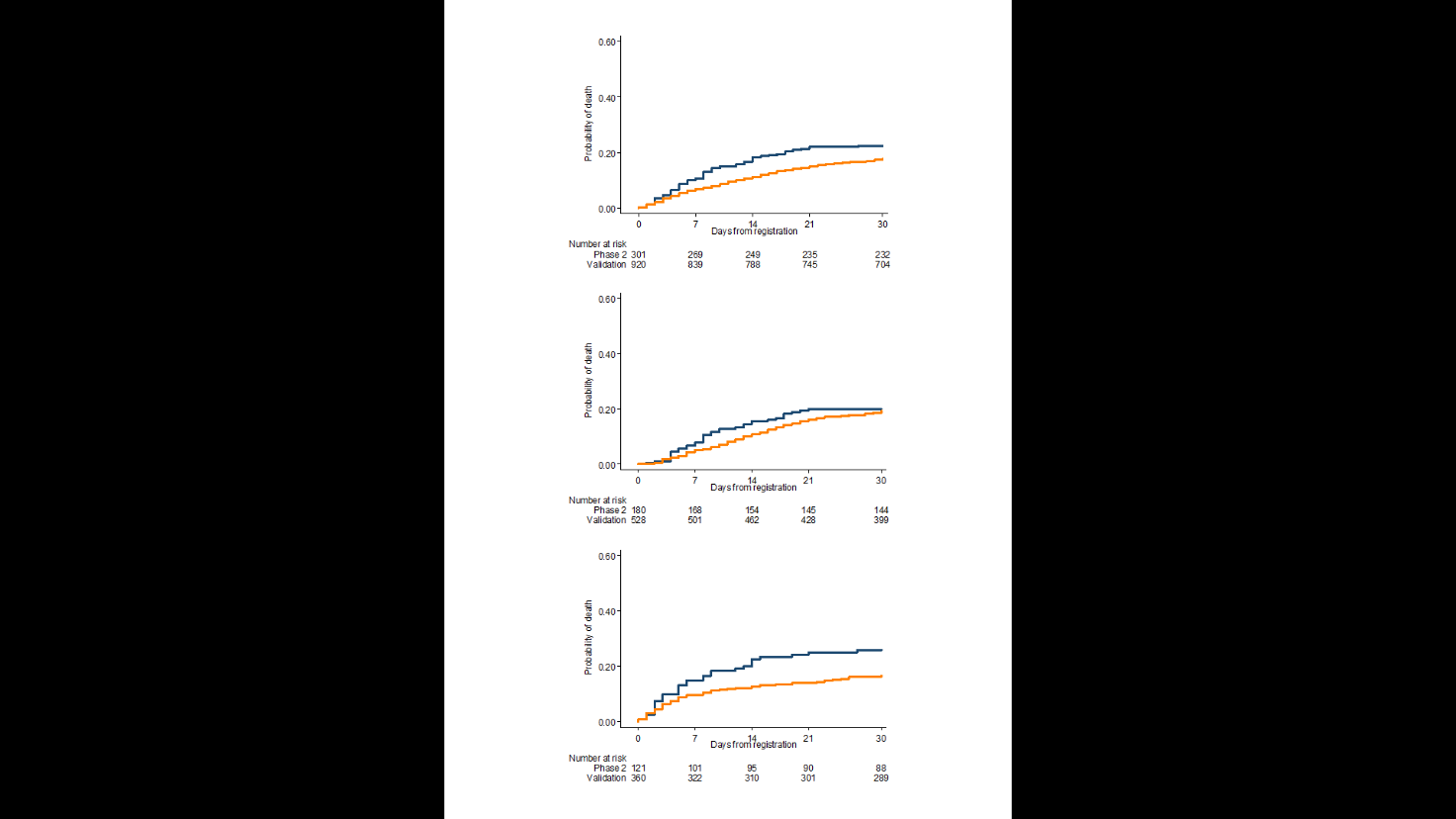


# **eTable 5. Lethality rates according to subgroups in ITT phase 2 and validation**

|  | **Phase 2** | |  | **Validation** | |
| --- | --- | --- | --- | --- | --- |
|  | **Lethality rate, % (95% CI)** | |  | **Lethality rate, % (95% CI)** | |
|  | **14 days** | **30 days** |  | **14 days** | **30 days** |
| **Tocilizumab administration** |  |  |  |  |  |
| ≤3 days from registration | 16.7 (10.9-24.0) | 19.6 (13.3-27.2) |  | 12.2 (9.2-15.9) | 19.7 (15.8-24.1) |
| ≥4 days from registration | 11.9 (4.0-25.6) | 21.4 (10.3-36.8) |  | 6.5 (2.8-12.4) | 21.0 (14.1-29.4) |
| Not treated | 22.7 (15.5-31.3) | 26.1 (18.4-34.9) |  | 12.7 (9.5-16.7) | 17.0 (13.2-21.4) |
| *P* | *0.23* | *0.47* |  | *0.16* | *0.53* |
| **Geographic area** |  |  |  |  |  |
| Lombardia | 20.1 (13.7-27.9) | 23.9 (16.9-32.0) |  | 14.7 (11.1-19) | 21.6 (17.2-26.5) |
| Veneto | 16.9 (8.8-28.3) | 20 (11.1-31.8) |  | 7.5 (1.6-20.4) | 19.4 (8.2-36.0) |
| Emilia Romagna | 13.5 (4.5-28.8) | 18.9 (8-35.2) |  | 12.8 (7.7-19.4) | 22.1 (15.6-29.9) |
| Other northern | - | - |  | 12.6 (6.5-21.5) | 14.3 (7.6-23.6) |
| Centre | 17.9 (7.5-33.5) | 25.6 (13-42.1) |  | 7.7 (4.3-12.6) | 15.3 (10.4-21.5) |
| South and Islands | 20.8 (7.1-42.2) | 20.8 (7.1-42.2) |  | 6.4 (2.6-12.8) | 12.1 (6.6-19.9) |
| *P* | *0.91* | *0.93* |  | *0.09* | *0.14* |
| **Age** |  |  |  |  |  |
| ≤60 | 2.5 (0.5-7.0) | 8.2 (4.0-14.6) |  | 4.5 (2.6-7.2) | 7.8 (5.2-11.1) |
| 61-70 | 19.8 (12.7-28.7) | 22.6 (15.1-31.8) |  | 7.4 (4.5-11.3) | 14.9 (10.7-20.0) |
| 71+ | 43.7 (31.9-56.0) | 46.5 (34.5-58.7) |  | 24.4 (19.4-30.0) | 35.7 (29.9-41.9) |
| *P* | ***<0.001*** | ***<0.001*** |  | ***<0.001*** | ***<0.001*** |
| **Gender** |  |  |  |  |  |
| Female | 18.6 (9.7-30.9) | 20.3 (11-32.8) |  | 9.9 (6.1-15.1) | 15.3 (10.4-21.3) |
| Male | 18.3 (13.6-23.8) | 22.9 (17.8-28.8) |  | 11.8 (9.5-14.5) | 19.3 (16.4-22.4) |
| *P* | *0.99* | *0.73* |  | *0.52* | *0.24* |
| **Body Mass Index** |  |  |  |  |  |
| Underweight/normal | 17.3 (9.6-27.8) | 22.7 (13.8-33.8) |  | 12.4 (8-18.1) | 21.5 (15.7-28.3) |
| Overweight/obese | 19.7 (14.2-26.2) | 22.4 (16.6-29.1) |  | 11.2 (8.6-14.3) | 18.2 (14.9-22) |
| *P* | *0.73* | *0.99* |  | *0.69* | *0.38* |
| **Smoking habit** |  |  |  |  |  |
| Never smoker | 19.2 (13.7-25.8) | 21.5 (15.7-28.3) |  | 11.0 (8.4-14.1) | 18.1 (14.8-21.9) |
| Previous or actual smoker | 21.6 (11.3-35.3) | 25.5 (14.3-39.6) |  | 11.7 (7.6-16.8) | 21.7 (16.2-28.1) |
| *P* | *0.84* | *0.57* |  | *0.89* | *0.29* |
| **Initial respiratory support** |  |  |  |  |  |
| Oxygen supplement | 16.6 (10.9-23.6) | 20 (13.8-27.4) |  | 7.5 (5.2-10.3) | 10.8 (8-14.0) |
| NIMV | 20.0 (12.8-28.9) | 22.9 (15.2-32.1) |  | 14.8 (11.2-18.9) | 22.9 (18.5-27.8) |
| IMV | 20.4 (10.2-34.3) | 28.6 (16.6-43.3) |  | 17.8 (10.5-27.3) | 40 (29.5-51.2) |
| *P* | *0.76* | *0.47* |  | ***0.001*** | ***<0.001*** |
| **PaO2/FiO2** |  |  |  |  |  |
| ≤100 | 27.3 (16.1-41.0) | 34.5 (22.2-48.6) |  | 15.6 (9.8-23.1) | 26.6 (19.1-35.3) |
| 101-200 | 14.5 (7.5-24.4) | 18.4 (10.5-29.0) |  | 7.9 (4.8-12.1) | 15.7 (11.3-21.1) |
| >200 | 2.6 (0.1-13.8) | 2.6 (0.1-13.8) |  | 5.7 (2.6-10.5) | 6.5 (3.1-11.5) |
| *P* | ***0.006*** | ***0.001*** |  | ***0.01*** | ***<0.001*** |
| **Heart disease** |  |  |  |  |  |
| None | 14.8 (10.4-20.1) | 18.8 (13.9-24.6) |  | 10.0 (7.9-12.6) | 17.1 (14.3-20.3) |
| Mild or worse | 25.8 (15.5-38.5) | 30.6(19.6-43.7) |  | 14 (8.9-20.6) | 22.6 (16.1-30.3) |
| *P* | *0.06* | *0.06* |  | *0.19* | *0.15* |
| **Hypertension** |  |  |  |  |  |
| None | 12.9 (7.8-19.6) | 17.1 (11.3-24.4) |  | 6.3 (4.2-9.1) | 11.2 (8.4-14.6) |
| Mild or worse | 21.4 (15-29.0) | 25.5 (18.6-33.4) |  | 15.8 (12.3-19.9) | 26.2 (21.7-31) |
| *P* | *0.06* | *0.11* |  | ***<0.001*** | ***<0.001*** |
| **Diabetes** |  |  |  |  |  |
| None | 16.3 (12.0-21.5) | 19.9 (15.2-25.4) |  | 10.3 (8.1-12.8) | 17.4 (14.6-20.6) |
| Mild or worse | 23.5 (10.7-41.2) | 32.4 (17.4-50.5) |  | 13.2 (8.0-20.1) | 21.8 (15.1-29.8) |
| *P* | *0.33* | *0.12* |  | *0.37* | *0.27* |
| **C-reactive protein** |  |  |  |  |  |
| ≤37 | 13.7 (8.0-21.3) | 16.2 (10.1-24.2) |  | 10.6 (7.5-14.4) | 18.1 (14-22.7) |
| >37 | 17.5 (11.2-25.5) | 20.8 (14.0-29.2) |  | 12.5 (9.1-16.7) | 19.6 (15.3-24.4) |
| *P* | *0.48* | *0.41* |  | *0.46* | *0.68* |
| **Concurrent steroids** |  |  |  |  |  |
| No | 20.8 (15.7-26.8) | 23.5 (18.1-29.7) |  | 12.8 (10.1-15.9) | 19.0 (15.7-22.6) |
| Yes | 4.8 (1.0-13.5) | 14.5 (6.9-25.8) |  | 7.9 (5.1-16.6) | 16.3 (12.2-21.2) |
|  | ***0.004*** | *0.16* |  | ***0.04*** | *0.39* |

# **eFigure 3. Estimated lethality rates at 14 and 30 days by baseline characteristics of patients in the validation ITT population. Red dash lines represent lethality rates under null hypotheses.**

#
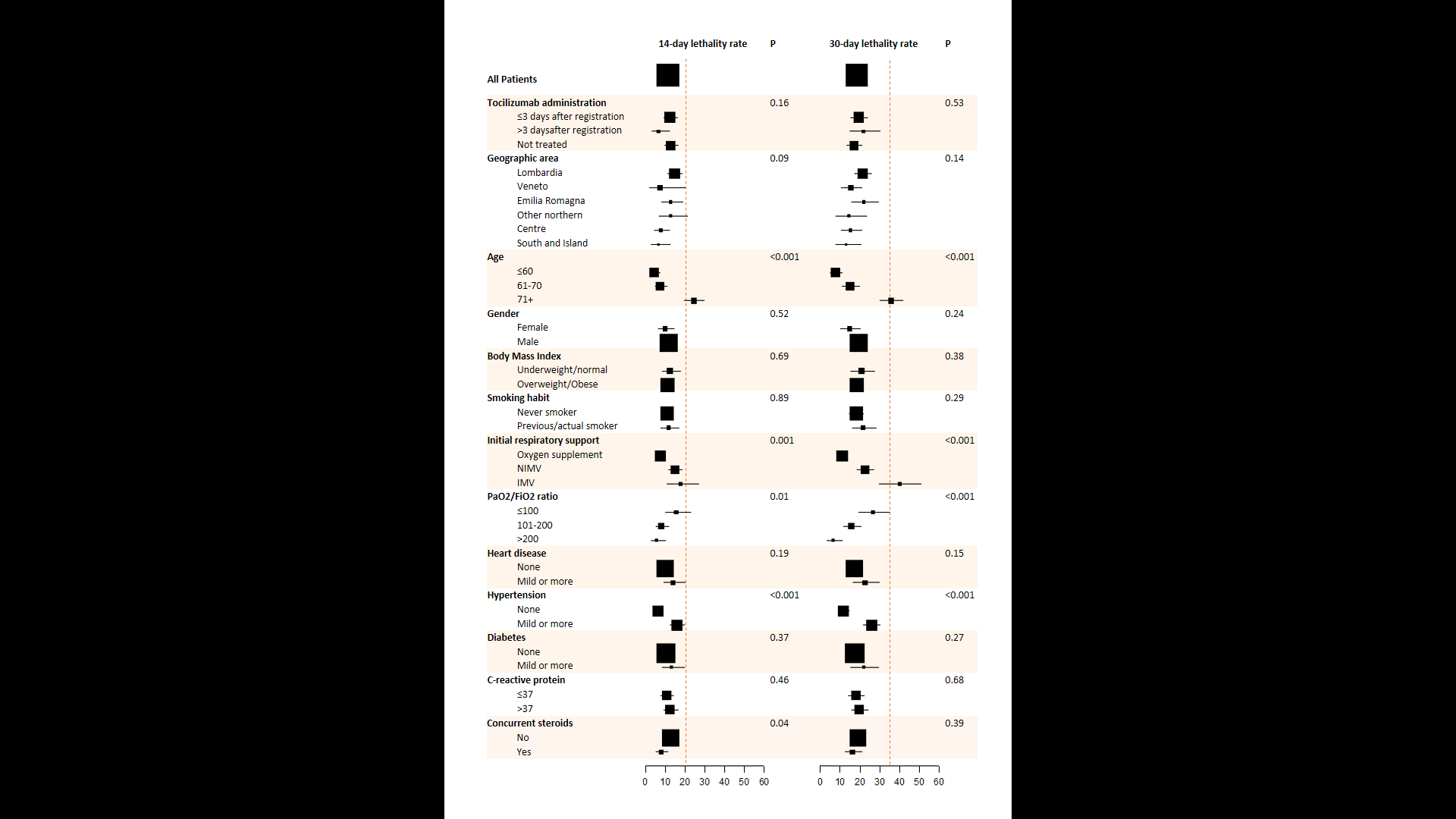
**eTable 6. Worst per patient adverse events reported after treatment with tocilizumab by CTCAE grade**

| **Category and terms** | **G0** | **G1** | **G2** | **G3** | **G4** | **G5** |
| --- | --- | --- | --- | --- | --- | --- |
|  | **no. (%)** | **no. (%)** | **no. (%)** | **no. (%)** | **no. (%)** | **no. (%)** |
| **Any adverse event** | **372 (59.2%)** | **45 (7.2%)** | **45 (7.2%)** | **55 (8.8%)** | **43 (6.8%)** | **68 (10.8%)** |
| **Blood lymphatic category** | **599 (95.4%)** | **8 (1.3%)** | **10 (1.6%)** | **9 (1.4%)** | **2 (0.3%)** | **0 (0.0%)** |
| anemia | 617 (98.2%) | 4 (0.6%) | 2 (0.3%) | 4 (0.6%) | 1 (0.2%) | 0 (0.0%) |
| **Cardiac category** | **605 (96.3%)** | **5 (0.8%)** | **7 (1.1%)** | **2 (0.3%)** | **3 (0.5%)** | **6 (1.0%)** |
| atrial fibrillation | 625 (99.5%) | 1 (0.2%) | 1 (0.2%) | 1 (0.2%) | 0 (0.0%) | 0 (0.0%) |
| atrial flutter | 627 (99.8%) | 0 (0.0%) | 1 (0.2%) | 0 (0.0%) | 0 (0.0%) | 0 (0.0%) |
| cardiac arrest | 623 (99.2%) | 0 (0.0%) | 0 (0.0%) | 0 (0.0%) | 0 (0.0%) | 5 (0.8%) |
| myocardial infarction | 627 (99.8%) | 0 (0.0%) | 0 (0.0%) | 0 (0.0%) | 1 (0.2%) | 0 (0.0%) |
| cardiac disorders other | 627 (99.8%) | 0 (0.0%) | 1 (0.2%) | 0 (0.0%) | 0 (0.0%) | 1 (0.2%) |
| **Gastrointestinal category** | **592 (94.3%)** | **13 (2.1%)** | **18 (2.9%)** | **3 (0.5%)** | **2 (0.3%)** | **0 (0.0%)** |
| diarrhea | 611 (97.3%) | 7 (1.1%) | 8 (1.3%) | 1 (0.2%) | 1 (0.2%) | 0 (0.0%) |
| mucositis oral | 624 (99.4%) | 1 (0.2%) | 3 (0.5%) | 0 (0.0%) | 0 (0.0%) | 0 (0.0%) |
| **General category** | **590 (93.9%)** | **14 (2.2%)** | **6 (1.0%)** | **4 (0.6%)** | **2 (0.3%)** | **12 (1.9%)** |
| fever | 609 (97.0%) | 13 (2.1%) | 4 (0.6%) | 1 (0.2%) | 1 (0.2%) | 0 (0.0%) |
| multi organ failure | 616 (98.1%) | 0 (0.0%) | 0 (0.0%) | 0 (0.0%) | 0 (0.0%) | 12 (1.9%) |
| **Hepatobliary category** | **627 (99.8%)** | **0 (0.0%)** | **0 (0.0%)** | **1 (0.2%)** | **0 (0.0%)** | **0 (0.0%)** |
| **Immune system category** | **625 (99.5%)** | **1 (0.2%)** | **1 (0.2%)** | **0 (0.0%)** | **1 (0.2%)** | **0 (0.0%)** |
| allergic reaction | 625 (99.5%) | 1 (0.2%) | 1 (0.2%) | 0 (0.0%) | 1 (0.2%) | 0 (0.0%) |
| **Infections category** | **597 (95.1%)** | **1 (0.2%)** | **8 (1.3%)** | **9 (1.4%)** | **5 (0.8%)** | **8 (1.3%)** |
| sepsis | 623 (99.2%) | 0 (0.0%) | 0 (0.0%) | 1 (0.2%) | 3 (0.5%) | 1 (0.2%) |
| infection other | 617 (98.2%) | 0 (0.0%) | 1 (0.2%) | 2 (0.3%) | 1 (0.2%) | 7 (1.1%) |
| **Investigations category** | **519 (82.6%)** | **34 (5.4%)** | **27 (4.3%)** | **34 (5.4%)** | **14 (2.2%)** | **0 (0.0%)** |
| ALT | 562 (89.5%) | 25 (4.0%) | 20 (3.2%) | 19 (3.0%) | 2 (0.3%) | 0 (0.0%) |
| AST | 571 (90.9%) | 22 (3.5%) | 18 (2.9%) | 16 (2.5%) | 1 (0.2%) | 0 (0.0%) |
| bilirubine increased | 621 (98.9%) | 1 (0.2%) | 2 (0.3%) | 3 (0.5%) | 1 (0.2%) | 0 (0.0%) |
| creatinine increased | 625 (99.5%) | 1 (0.2%) | 0 (0.0%) | 0 (0.0%) | 2 (0.3%) | 0 (0.0%) |
| lymphocyte count decreased | 625 (99.5%) | 1 (0.2%) | 2 (0.3%) | 0 (0.0%) | 0 (0.0%) | 0 (0.0%) |
| neutrophil count decreased | 609 (97.0%) | 3 (0.5%) | 6 (1.0%) | 7 (1.1%) | 3 (0.5%) | 0 (0.0%) |
| platelet count decreased | 610 (97.1%) | 2 (0.3%) | 7 (1.1%) | 6 (1.0%) | 3 (0.5%) | 0 (0.0%) |
| investigations other | 625 (99.5%) | 2 (0.3%) | 0 (0.0%) | 1 (0.2%) | 0 (0.0%) | 0 (0.0%) |
| **Metabolism category** | **614 (97.8%)** | **6 (1.0%)** | **2 (0.3%)** | **3 (0.5%)** | **3 (0.5%)** | **0 (0.0%)** |
| **Muscoloskeletal category** | **626 (99.7%)** | **0 (0.0%)** | **1 (0.2%)** | **0 (0.0%)** | **0 (0.0%)** | **1 (0.2%)** |
| **Nervous system category** | **614 (97.8%)** | **4 (0.6%)** | **4 (0.6%)** | **4 (0.6%)** | **1 (0.2%)** | **1 (0.2%)** |
| intracranial hemorrhage | 627 (99.8%) | 0 (0.0%) | 0 (0.0%) | 0 (0.0%) | 0 (0.0%) | 1 (0.2%) |
| **Renal category** | **616 (98.1%)** | **3 (0.5%)** | **3 (0.5%)** | **3 (0.5%)** | **2 (0.3%)** | **1 (0.2%)** |
| acute kidney injury | 624 (99.4%) | 0 (0.0%) | 0 (0.0%) | 2 (0.3%) | 1 (0.2%) | 1 (0.2%) |
| **Respiratory category** | **535 (85.2%)** | **4 (0.6%)** | **13 (2.1%)** | **10 (1.6%)** | **31 (4.9%)** | **35 (5.6%)** |
| ARDS | 619 (98.6%) | 0 (0.0%) | 0 (0.0%) | 0 (0.0%) | 6 (1.0%) | 3 (0.5%) |
| dyspnea | 592 (94.3%) | 3 (0.5%) | 5 (0.8%) | 9 (1.4%) | 19 (3.0%) | 0 (0.0%) |
| hypoxia | 622 (99.0%) | 0 (0.0%) | 3 (0.5%) | 2 (0.3%) | 1 (0.2%) | 0 (0.0%) |
| pneumonitis | 624 (99.4%) | 0 (0.0%) | 0 (0.0%) | 1 (0.2%) | 3 (0.5%) | 0 (0.0%) |
| pneumothorax | 625 (99.5%) | 1 (0.2%) | 1 (0.2%) | 1 (0.2%) | 0 (0.0%) | 0 (0.0%) |
| respiratory failure | 585 (93.2%) | 0 (0.0%) | 0 (0.0%) | 0 (0.0%) | 13 (2.1%) | 30 (4.8%) |
| respiratory other | 620 (98.7%) | 1 (0.2%) | 0 (0.0%) | 4 (0.6%) | 1 (0.2%) | 2 (0.3%) |
| **Vascular category** | **606 (96.5%)** | **1 (0.2%)** | **3 (0.5%)** | **10 (1.6%)** | **4 (0.6%)** | **4 (0.6%)** |
| hypertension | 615 (97.9%) | 1 (0.2%) | 3 (0.5%) | 7 (1.1%) | 2 (0.3%) | 0 (0.0%) |
| thromboembolic event | 621 (98.9%) | 0 (0.0%) | 0 (0.0%) | 3 (0.5%) | 1 (0.2%) | 3 (0.5%) |
| vascular disorder other | 627 (99.8%) | 0 (0.0%) | 0 (0.0%) | 0 (0.0%) | 0 (0.0%) | 1 (0.2%) |

# **eTable 7. Multivariable logistic regression**

(patients receiving tocilizumab more than three days after registration are excluded)

|  | **14 days (143 events/1002 patients)** | | |  | **30 days (191 events/980 patients)** | | |
| --- | --- | --- | --- | --- | --- | --- | --- |
|  | **Beta coefficient** | **OR of death (95% CI)** | **P** |  | **Beta coefficient** | **OR of death (95% CI)** | **P** |
| **Age** |  |  | <0.001 |  |  |  | <0.001 |
| ≤60 |  | Reference |  |  |  | Reference |  |
| 61-70 |  | 2.83 (1.55-5.18) |  |  |  | 2.31 (1.41-3.80) |  |
| 70+ |  | 12.38 (6.99-21.92) |  |  |  | 9.51 (5.92-15.29) |  |
| **Gender** |  |  | 0.52 |  |  |  | 0.32 |
| Female |  | Reference |  |  |  | Reference |  |
| Male |  | 1.18 (0.72-1.93) |  |  |  | 1.26 (0.80-1.98) |  |
| **PaO2/FiO2 ratio** |  |  | 0.02 |  |  |  | 0.001 |
| ≤100 |  | Reference |  |  |  | Reference |  |
| 101-200 |  | 0.48 (0.26-0.89) |  |  |  | 0.40 (0.28-0.84) |  |
| >200 |  | 0.33 (0.14-0.75) |  |  |  | 0.23 (0.11-0.50) |  |
| Missing or not assessed |  | 0.75 (0.43-1.31) |  |  |  | 0.68 (0.41-1.12) |  |
| **Geographic area** |  |  | 0.24 |  |  |  | 0.33 |
| Lombardia |  | Reference |  |  |  | Reference |  |
| Veneto |  | 0.58 (0.26-1.29) |  |  |  | 0.72 (0.36-1.45) |  |
| Emilia Romagna |  | 1.07 (0.57-1.98) |  |  |  | 1.20 (0.70-2.06) |  |
| Other Northern Regions |  | 0.87 (0.39-1.93) |  |  |  | 0.64 (0.30-1.35) |  |
| Centre |  | 0.54 (0.29-1.00) |  |  |  | 0.67 (0.39-1.15) |  |
| South and Islands |  | 0.55 (0.25-1.23) |  |  |  | 0.69 (0.34-1.39) |  |
| **Concurrent steroids** |  |  | <0.001 |  |  |  | 0.01 |
| Not used |  | Reference |  |  |  | Reference |  |
| Used |  | 0.36 (0.21-0.62) |  |  |  | 0.62 (0.40-0.95) |  |
| Missing |  | 1.55 (0.74-3.26) |  |  |  | 1.81 (0.90-3.63) |  |
| **Population** |  |  | 0.01 |  |  |  | 0.28 |
| Phase 2 |  | Reference |  |  |  | Reference |  |
| Validation |  | 0.55 (0.35-0.88) |  |  |  | 0.79 (0.52-1.21) |  |
| **Tocilizumab** |  |  |  |  |  |  |  |
| Not administered | Reference |  |  |  | Reference |  |  |
| Administered | -1.009 |  |  |  | -0.690 |  |  |
| **Respiratory support** |  |  |  |  |  |  |  |
| Oxygen | Reference |  |  |  | Reference |  |  |
| NIMV | 0.254 |  |  |  | 0.333 |  |  |
| IMV | -0.024 |  |  |  | 1.149 |  |  |
| **Tocilizumab*Respiratory support** |  |  | 0.03 |  |  |  | 0.08 |
| Oxygen | Reference |  |  |  | Reference |  |  |
| NIMV | 1.026 |  |  |  | 0.938 |  |  |
| IMV | 1.522 |  |  |  | 0.710 |  |  |

# **eFigure 4. Relative (Odds Ratio of death, panel A) and absolute (difference in lethality rate, panel B) measures of tocilizumab effect by required respiratory support at 14 and 30 days.**

#
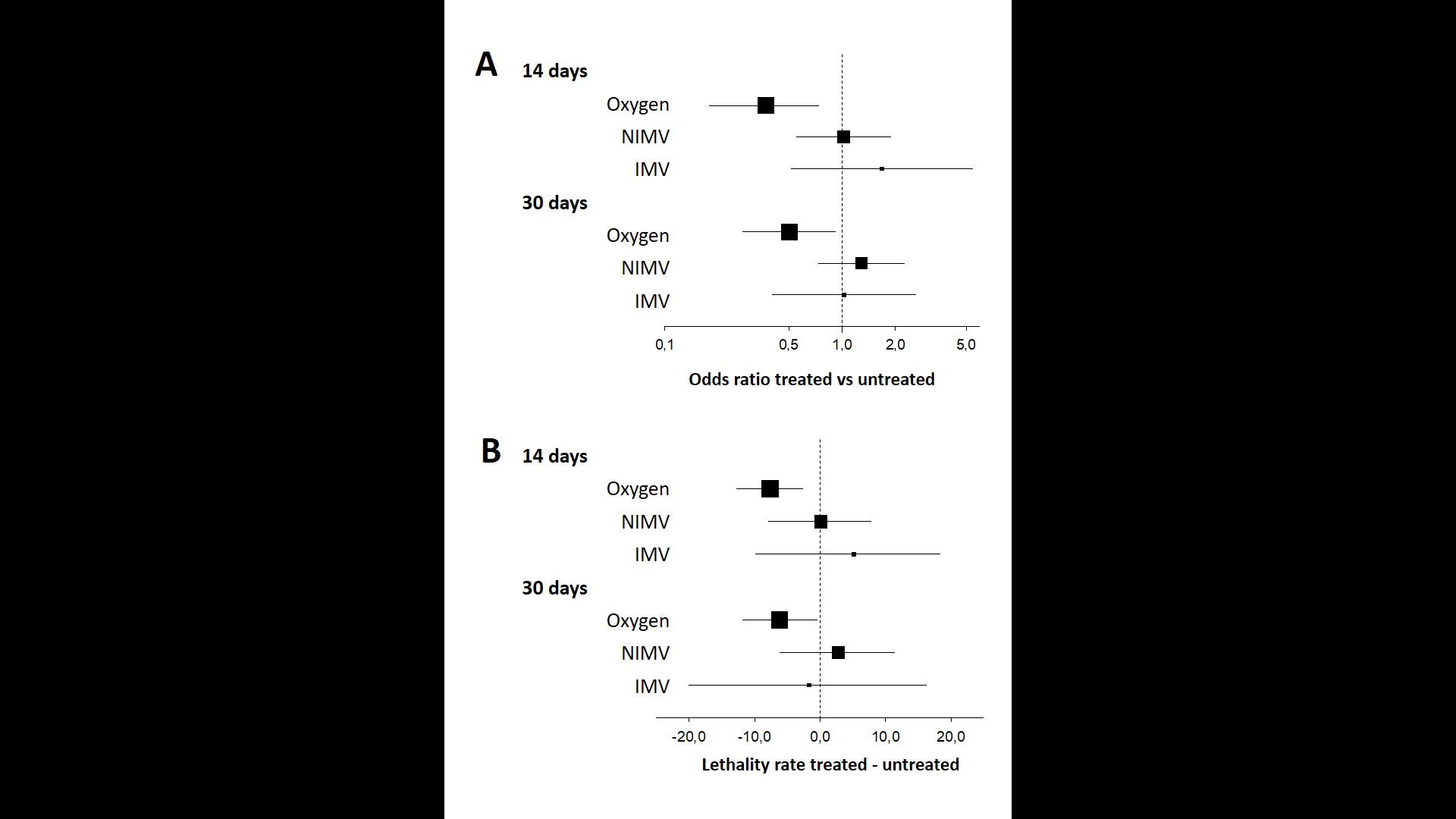
**List of participating centres and Co-Investigators**

**TOCIVID-19 Investigators**

- Istituto Nazionale Tumori, IRCCS, Fondazione G. Pascale, Napoli - Clinical Trials Unit: Francesco Perrone, Maria Carmela Piccirillo, Clorinda Schettino, Adriano Gravina, Piera Gargiulo, Claudia Cardone, Laura Arenare; Melanoma And Cancer Immunotherapy And Developmental Therapeutics Unit: Paolo Antonio Ascierto, Maria Grazia Vitale, Claudia Trojaniello, Marco Palla; Direction: Attilio Antonio Montano Bianchi, Gerardo Botti, Gianfranco De Feo, Leonardo Miscio.
- Università degli Studi della Campania Luigi Vanvitelli, Dipartimento di Salute Mentale e Medicina Preventiva; Ciro Gallo, Paolo Chiodini.
- IRCCS Policlinico San Donato - Milano: Laurenzia Ferraris, Massimiliano M. Marrocco-Trischitta, Marco Froldi, Lorenzo Menicanti, Maria Teresa Cuppone, Giulia Gobbo, Chiara Baldessari, Vincenzo Valenti, Serenella Castelvecchio, Federica Poli, Francesca Giacomazzi, Rosangela Piccinni, Maria Laura Annnunziata, Andrea Biondi, Cecilia Bussolari, Manuel Mazzoleni, Andrea Giachi, Annalisa Filtz, Arianna Manini, Enrico Poletti, Federico Masserini, Francesco Conforti, Gianfranco Gaudiano, Vittoria Favero, Alice Moroni, Tommaso Viva, Fabiana Fancoli, Davide Ferrari, Dario Niro, Marco Resta, Andrea Ballotta, Marco Dei Poli, Marco Ranucci.
- ASST Papa Giovanni XXIII – Bergamo: Diego Ripamonti, Francesca Binda, Alessandra Tebaldi, Giuseppe Gritti, Luisa Pasulo, Leonardo Gaglio, Roberto Del Fabbro, Leonardo Alborghetti.
- ASST Monza – Monza: Paolo Bonfanti, Nicola Squillace, Giulia Giustinetti, Paola Columpsi, Marina Cazzaniga, Serena Capici, Luca Sala, Riccardo Di Sciacca, Giacomo Mosca, Maria Rosa Pirozzi.
- ASST degli Spedali Civili di Brescia e Università di Brescia - Brescia: Francesco Castelli, Maria Lorenza Muiesan, Franco Franceschini, Aldo Roccaro, Massimo Salvetti, Anna Paini, Luciano Corda, Chiara Ricci, Lina Tomasoni, Paola Nasta, Silvia Lorenzotti, Silvia Odolini, Emanuele Focà, Eugenia Quiros Roldan, Marco Metra, Stefano Magrini, Paolo Borghetti, Nicola Latronico, Simone Piva, Matteo Filippini, Gabriele Tomasoni, Francesco Zuccalà, Sergio Cattaneo, Francesco Scolari, Nicola Bossini, Mario Gaggiotti, Martina Properzi.
- Ospedale Santa Maria Goretti – Latina: Miriam Lichtner, Emanuela Del Giudice, Raffaella Marocco, Anna Carraro, Cosmo Del Borgo, Raffaella Marocco, Valeria Belvisi, Tiziana Tieghi, Margherita De Masi, Paola Zuccalà, Paolo Fabietti, Angelo Vetica, Vito Sante Mercurio, Anna Carraro, Laura Fondaco, Blerta Kertusha, Ambrogio Curtolo, Emanuela Del Giudice, Riccardo Lubrano, Maria Gioconda Zotti, Antonella Puorto, Marcello Ciuffreda, Antonella Sarni, Gabriella Monteforte, Domenico Romeo, Emanuela Viola, Carla Damiani, Antonietta Barone, Barbara Mantovani, Daniela Di Sanzo, Vincenzo Gentili, Massimo Carletti, Massimo Aiuti, Andrea Gallo, Piero Giuseppe Meliante, Salvatore Martellucci, Oliviero Riggio, Vincenzo Cardinale, Lorenzo Ridola, Maria Consiglia Bragazzi, Stefania Gioia, Emiliano Valenzi, Camilla Graziosi, Niccolò Bina, Martina Fasolo, Silvano Ricci, Maria Teresa Gioacchini, Antonella Lucci, Luisella Corso, Daniela Tornese, Parni Nijhawan, Francesco Equitani, Carmine Cosentino, Marcello Palladino, Frida Leonetti, Gaetano Leto, Camillo Gnessi, Giuseppe Campagna, Roberto Cesareo, Francesca Marrocco, Giuseppe Straface, Alessandra Mecozzi, Lidia Cerbo, Valentina Isgrò, Sergio Parrocchia, Giuseppe Visconti, Giorgio Casati.
- AOU di Parma - Parma: Carlo Calzetti, Alarico Ariani, Lorenzo Donghi.
- AOUI di Verona - Verona: Nicola Duccio Salerno, Evelina Tacconelli, Marco Bertoldi, Paolo Cattaneo, Lorenza Lambertenghi, Leonardo Motta, Luca Omega.
- Humanitas Gavazzeni – Bergamo: Giovanni Albano.
- AORN Dei Colli – Napoli: Roberto Parrella, Fiorentino Fraganza, Luigi Atripaldi, Vincenzo Montesarchio, Francesco Scarano, Annunziata De Rosa, Amalia Buglione, Sabrina Lavoretano, Gianfranco Gaglione, Mario De Marco, Vincenzo Sangiovanni, Francesco Maria Fusco, Rosaria Viglietti, Elio Manzillo, Carolina Rescigno, Raffaella Pisapia, Giulia Plamieri, Alberto Maraolo, Giosuè Calabria, Mario Catalano, Giuseppe Fiorentino, Anna Annunziata, Giorgio Polistina, Pasquale Imitazione, Mariano Mollica, Vincenzo Esposito, Maurizio D’Abraccio, Rodolfo Punzi, Vincenzo Bianco, Costanza Sbreglia.
- Azienda Ospedaliera Umberto I – Siracusa: Rosa Fontana Del Vecchio, Alessandro Bordonali, Antonina Franco.
- Arcispedale Santa Maria Nuova IRCCS - Reggio Emilia: Carlo Salvarani, Marco Massari, Giovanni Dolci, Pierpaolo Salsi, Matteo Fontana.
- ASST di Cremona - Cremona: Giuseppe Virzì, Calderone Ornella, Alfredo Molteni.
- Azienda Ospedaliera San Salvatore – Pesaro: Silvia Gennarini, Umberto Gnudi, Maria Anastasia Ricci, Giancarlo Titolo, Giulio Mensi, Pietro Vuotto, Beatrice Gasperini, Mauro Mancini, Zeno Pasquini.
- Ospedale Bassini - Cinisello Balsamo: Paolo Spanu, Stefano Clementi, Simona Pierini, Daniela Bokor, Daniela Gori, Morena Ciofetti, Marina Caimi, Laura Bettazzi, Elisabetta Allevi, Silvia Furiani, Chiara Capitanio, Bernardino Mastropasqua, Claudio Fara, Grazia Pulitanò, Jun Sebastian Matsuno, Francesca Della Porta, Viola Dolfini, Nebiat Balei Beyene.
- ASST Degli Spedali Civili Di Brescia - Brescia: Michela Bezzi, Mauro Novali.
- AOU di Bologna - Bologna: Pierluigi Viale, Sara Tedeschi, Renato Pascale.
- Policlinico S. Matteo - Pavia: Raffaele Bruno, Alessandro Di Filippo, Michele Sachs, Tiberio Oggionni, Michele Di Stefano, Caterina Mengoli.
- Ospedale di Conegliano - Conegliano: Cesarina Facchini, De Nardo Daniele.
- Azienda Ospedaliera San Salvatore – Pesaro: Gabriele Frausini, Luciano Mucci, Silvia Tedesco, Rita Girolimetti, Elena Manfredini, Anna Maria Di Carlo, Emma Espinosa, Donatella Dennetta.
- AOU di Parma - Parma: Andrea Ticinesi, Tiziana Meschi, Antonio Nouvenne.
- Azienda Ospedaliera Ordine Mauriziano – Torino: Norbiato Claudio, Francesco Vitale, Marta Saracco.
- Ospedale Guglielmo Da Saliceto – Piacenza: Mauro Codeluppi, Elisa Fronti, Patrizia Ferrante.
- Ospedale di Fermo - Fermo: Giorgio Amadio Nespola.
- AOU di Perugia - Perugia: Daniela Francisci, Andrea Tosti.
- Casa Sollievo Della Sofferenza - San Giovanni Rotondo: Cristiano Matteo Carbonelli, Antonio Greco, Maria Giulia Tinti.
- Fondazione Poliambulanza Istituto Ospedaliero – Brescia: Roberto Stellini, Camilla Appiani, Piera Reghenzi.
- Ospedale Morgagni-Pierantoni - Forlì: Venerino Poletti, Claudia Ravaglia.
- Ospedale Area Aretina Nord - Arezzo: Danilo Tacconi, Costanza Malcontenti.
- AOU “Maggiore della Carità” - Novara: Pier Paolo Sainaghi, Raffaella Landi, Veronica Vassia, Eleonora Rizzi, Mattia Bellan, Antonella Rossati, Luigi Castello
- Policlinico Umberto I – Roma: Claudio Maria Mastroianni, Gianluca Russo.
- Presidio Ospedaliero di Jesolo - Jesolo: Toffoletto Fabio, Francesco Saverio Serino, Lucio Brollo, Elena Momesso, Maria Luisa Turati.
- ASST Santi Paolo e Carlo – Milano: Antonella D'arminio Monforte, Giulia Marchetti.
- Ospedale Civile di Guastalla - Guastalla: Fabrizio Boni, Elisabetta Teopompi, Chiara Trenti, Luca Boracchia, Enrica Minelli, Matteo Fontana, Giulia Ghidoni, Anaflorina Matei, Andrea Caruso.
- AO Ospedali Riuniti Villa Sofia e Cervello – Palermo: Giuseppe Arcoleo, Gaetana Camarda, Filippo Catalano, Mario Spatafora.
- Ospedale Sacra Famiglia, Fatebenefratelli – Erba: Donato Bettega.
- AOU Policlinico Tor Vergata – Roma: Massimo Andreoni, Elisabetta Teti, Loredana Sarmati, Andrea Di Lorenzo, Mariagrazia Celeste.
- Ospedali Riuniti Padova Sud - Padova: Fabio Baratto, Jacopo Monticelli, Pietro Criveller.
- Ospedale San Paolo – Savona: Antonini Andrea, Anselmo, Riccio.
- ASST Spedali Civili Di Brescia - Brescia: Maurizio Castellano, Carlo Cappelli, Federica Corvini, Barbara Zanini.
- ASST Spedali Civili Di Brescia, Presidio Ospedaliero Gardone – Brescia: Massimo Crippa, Maurizio Ronconi, Raffaella Costa, Silvia Casella, Loretta Brentana.
- Ospedale Civile e Ospedale Dell'Angelo - Mestre: Livio Bernardi, Andrea Frascati, Sandro Panese, Fabio Presotto, Lucio Michieletto, Cristina Bernardi.
- Ospedale Santa Maria Delle Croci – Ravenna: Maurizio Fusar.
- Presidio Ospedaliero di Cesena - Cesena: Vanni Agnoletti, Martina Farina, Russo.
- AOU Careggi – Firenze: Federico Lavorini, Roberta Ginanni.
- Istituto Nazionale Malattie Infettive INMI L. Spallanzani, IRCCS – Roma: Fabrizio Palmieri, Silvia Mosti.
- Casa Di Cura Beato Palazzolo – Bergamo: Angelo Amaglio, Alessandra Cattaneo.
- Istituto Clinico S. Ambrogio Spa – Milano: Silvia Cirri, Andrea Montisci, Chiara Gallazzi, Daniele Cosseta, Barabara Baronio, Lorenzo Rampa.
- AO Sant’Anna e San Sebastiano – Caserta: Paolo Maggi, Vincenzo Messina.
- Arcispedale Santa Maria Nuova IRCCS - Reggio Emilia: Emanuele Alberto Negri, Chiara Trenti.
- Ospedale Generale Provinciale – Macerata: Marialma Berlendis, Maria Cecilia Sabatti.
- Azienda Ospedaliera S.Maria - Terni: Michele Palumbo.
- ASST Ovest Milanese – Milano: Antonino Mazzone, Paola Faggioli.
- Ospedale Bellaria – Bologna: Linda Bussini, Giacomo Fornaro, Francesca Volpato.
- Ospedale Maria Vittoria – Torino: Daniele Imperiale, Emilpaolo Manno, Enrico Ferreri, Domenico Martelli, Andrea Verhovez, Silvia Giorgis, Luciana Faccio, Rachele Delli Quadri, Cristina Negro.
- Ospedale Giovanni Bosco – Torino: Marcella Converso, Francesca Bosco.
- ASST Desenzano Del Garda – Gavardo: Silvia Amadasi, Paolo Prandini, Silvia Cocchi.
- Azienda ULSS 6 – Vicenza: Vinicio Manfrin, Veronica Del Punta.
- PO Sant’Elia – Caltanissetta: Giovanni Mazzola, Giuseppe Sportato.
- Ospedale Ca’ Foncello – Treviso: Micaela Romagnoli.
- Ospedale Infermi - Rimini: Francesco Cristini, Francesca Facondini, Tiziana Perin, Andrea Boschi.
- AOU di Modena - Modena: Cristina Mussini, Marianna Meschiari, Giovanni Guaraldi.
- Presidio Ospedaliero San Luca – Lucca: Sara Modica, Sara Moneta, Daniela Boccalatte, Clara Ricci, Valentina Marchetti.
- ASST Desenzano Del Garda - Ospedale Di Manerbio: Silvia Amadasi, Gabriele Ebbreo, Michael Dalè, Paolo Tura.
- AO Spedali Civili, PO Montichiari – Brescia: Damiano Rizzoni, Gianluca Edoardo Mario Boari, Silvia Bonetti.
- Ospedale San Liberatore di Atri - Teramo: Enrico Marini, Italiani Daniele.
- ASST Dei Sette Laghi – Varese: Paolo Antonio Grossi, Nichola Walter Delfrate.
- Ospedale Aziendale Di Bressanone - Bressanone: Othmar Bernhart, Gilbert Spizzo, Klaus Mahlknecht, Thomas Volkl.
- Ospedale San Jacopo - USL Toscana Centro - Pistoia: Massimo Antonio Di Pietro, Michele Trezzi, Cecilia Monacci.
- Ospedale di Rovigo - Rovigo: Adriano Peris, Manuela Bonizzoli.
- Ospedale Guglielmo Da Saliceto – Piacenza: Luigi Cavanna, Carlo Moroni, Elisa Maria Stroppa, Alessandra Manini.
- ASST Garda - Ospedale Civile La Memoria - Gavardo: Maria Cristina Savio, Francesca Gatti, Clara Bartolaminelli.
- Istituto Nazionale Malattie Infettive INMI L. Spallanzani, IRCCS – Roma: Nicola Petrosillo, Davide R. Donno, Fabrizio Taglietti, Simone Topino, Pierangelo Chinello, Vincenzo Galati.
- Istituto Nazionale Malattie Infettive INMI L. Spallanzani, IRCCS – Roma: Gianpiero D'offizi, Chiara Taibi.
- Ospedale Fiorentino – Firenze: Barbara Cimolato, Federico Moroni, Nicolas Palagano, Lorenzo Pelagatti, Seravalle Cristiana, Giancarlo Landini.
- Azienda Ospedaliera S. G. Moscati – Avellino: Maria Amitrano, Mariangela Raimondo, Sara Mangiacapra, Annamaria Romano, Mariangela Atteno.
- Ospedale S.M. Annunziata - AUSL Toscana Centro - Grassina: Pierluigi Blanc, Lorenzo Roberto Suardi, Carlo Pallotto
- Ospedale F. Spaziani – Frosinone: Katia Casinelli, Ilaria Uccella.
- Ospedale S. Giuseppe – Milano: Sergio Harari, Antonella Caminati.
- Ospedale Amedeo Di Savoia – Torino: Filippo Lipani, Giovanni Di Perri, Andrea Calcagno, Guido Calleri, Chiara Montrucchio, Anna Maria Caputo.
- Presidio Ospedaliero S.Maria Del Carmine –Rovereto: Susanna Cozzio, Livia Delle Donne.
- AO Policlinico Ospedale S. Martino – Genova: Matteo Bassetti, Mikulska Malgorzata, Laura Ambra Nicolini, Chiara Russo, Chiara Sepulcri, Sabrina Beltramini, Federica Mina.
- ASST Grande Ospedale Metropolitano Niguarda – Milano: Massimo Puoti, Anna Gandino, Thomas Langer, Federico D'amico.
- ASST Spedali Civili Di Brescia - Brescia: Marialma Berlendis, Chiara Rocchetti, Francesca Cettolo.
- Presidio Ospedaliero Aziendale, AUSL Parma - Parma: Frausini Gabriele, Pietro Bocchi.
- Ospedale Pavullo nel Frignano – Modena: Giorgio Cioni, Cinzia Cappi.
- AOU Città della Salute e della Scienza - Ospedale Le Molinette – Torino: Silvia Corcione, Francesco Giuseppe De Rosa, Silvia Scabini, Francesca Canta, Simone Mornese Pinna, Anna Pensa.
- Ospedale San Giuseppe - AUSL Toscana Centro - Empoli: Pierluigi Blanc, Lorenzo Roberto Suardi, Carlo Pallotto.
- Azienda Ospedaliera Sant’Andrea – Roma: Monica Rocco, Maria Teresa Cirasa.
- AOU Careggi – Firenze: Michele Spinicci, Jessica Mencarini, Lorenzo Zammarchi.
- Presidio Ospedaliero Unificato - San Remo: Cenderello Giovanni, Katiuscia Sciolè.
- Presidio Ospedaliero Santa Maria della Misericordia – Udine: Flavio Bassi.
- Casa Di Cura S. Rita – Milano: Michele Bianchi, Sillia Frigerio.
- Ospedale F. Spaziani – Frosinone: Sandra Spaziani, Antonia Nucera.
- AO Luigi Sacco - Milano: Giuliano Rizzardini, Maria Vittoria Cossu, Marco Antivalle.
- AOU Policlinico Vittorio Emanuele – Catania: Giuseppe Carpinteri.
- Presidio Ospedaliero Riuniti - Reggio Calabria: Sebastiano Macheda, Demetrio Labate.
- ASST Grande Ospedale Metropolitano Niguarda – Milano: Maurizio Bottiroli.
- Ospedale V. Fazzi - Lecce: Anacleto Romano.
- Ospedale Generale Regionale – Bolzano: Elke Maria Erne, Zocchetti Cristina.
- Ospedale Mazzini – Teramo: Valeria Di Biase.
- ASST di Cremona - Cremona: Fabio Malberti, Alfredo Molteni.
- ASST degli Spedali Civili di Brescia - Brescia: Giovanni Montani, Paolo Poisa, Daniela Bettini.
- Policlinico Universitario A. Gemelli – Roma: Roberto Cauda, Arturo Ciccullo.
- Ospedale Sacro Cuore Don Calabria - Negrar (Verona): Niccolò Riccardi, Andrea Angheben.
- Ospedale Valduce Dous – Como: Mauro Turrini, Raffaella Clerici, Angelo Gardellini, Luigi Liparulo, Tizana Rossini.
- PO SS. Annunziata – Chieti: Claudio Ucciferri, Francesco Cipollone, Jacopo Vecchiet.
- Ospedale S.G. Moscati – ASL Taranto - Taranto: Andrea Nico, Lorenzo Marra, Armando Leone, Antonia Sdanganelli, Giuseppe Antonio Palmiotti, Giancarlo D’Alagni.
- AOU Opsedali Riuniti – Foggia: Teresa Antonia Santantonio, Sergio Lo Caputo, Irene Bottalico.
- AO Sant’Anna e San Sebastiano – Caserta: Antonio Ponticiello, Felicia Di Perna.
- Ospedale Ca’ Foncello – Treviso: Enrico Bernardi, Angela Beltrame, Stefania Bravi, Marco David, Paola Bernardi.
- Ospedale G.Tatarella - Cerignola (Foggia): Dario Galante.
- ASST Franciacorta - Presidio Ospedaliero di Iseo: Maria Cristina Uccelli, Katiela Prestini, Monica Drera, Enea Zini, Alessio Peregrinelli, Laura Blanzuoli.
- Ospedale di Mondovì - ASL CN1 – Cuneo: Valentina Benedetti, Rovberta Calvi, Nadia Scaglione, Gabriella Nallino.
- IRCCS Istituto Ortopedico Galeazzi Spa – Milano: Maurizio Bonazzi, Tiziano Crespi, Tiziana Masolin.
- Ospedale Delmati - Sant Angelo Lodigiano: Angelo Regazzetti, Maria Chiara Cerri, Elena Maffezzini, Manuela Piazza, Claudia Papetti, Claudia De Filippi, Elena Roveda, Giuseppe Cipolla, Mariano Scozzafava, Monica Crepaldi, Sonia Henchi, Nicolò Vanoni, Alice Repossi, Monia Vezzoli, Eva Scorletti, Orietta Perugini, Simone Marino Pasini, Veronica Pacetti, Luisella Ferrari, Giovanna Attilia de Paduanis, Sara del Duca, Francesca dell’Ara, Alessandra Brocchieri, Guja Minoja, Enrico Storti, Loredana Pitagora, Isabella Costa, Fanny Delfanti, Matteo Orlandi.
- AO Ospedale Niguarda Ca Granda – Milano: Ruggero Ruggeri, Lorenzoa Ruggieri.
- AO S. Giovanni Bosco – Torino: Sergio Livigni, Daniela Silengo.
- AO Ospedale di Circolo e Fondazione Macchi – Varese: Walter Ageno.
- Ospedale Bolognini – Seriate: Luciano Pedrini.
- Ospedale Sant’Andrea - La Spezia: Stefania Artiol.
- Ospedale Di Senigallia - Senigallia: Laura Morbidoni.
- ASST di Mantova - Mantova: Giuseppe De Donno, Viviana Ravagnani, Francesco Inglese.
- Ospedale Ca’ Foncello – Treviso: Pier Giorgio Scotton.
- Ospedale Civile Di Saluzzo – ASL CN1 – Cuneo: Paolo Costantini, Maurizio Delucchi.
- AOU di Modena - Modena: Enrico Clini.
- Ospedale Di Senigallia - Senigallia: Andrea Ansuini.
- Ospedale San Bassiano - Bassano Del Grappa: Baiocchi Marco, Lain Giuseppe.
- Presidio Ospedaliero Aziendale AUSL Parma – Fidenza: Brianti Vincenzo, Gianni Rastelli.
- AOU di Padova - Padova: Andrea Doria, Andrea Vianello, Anna Maria Cattelan, Sara Bindoli, Mara Felicietti.
- ASST di Crema - Crema: Ciro Canetta, Alessandro Scartabellati, Silvia Accordino.
- PO Sant'Ottone Frangipane - Ariano Irpino: Maurizio Ferrara, Livio Cocco, Fernanda Cirillo, Erminio Pace, Monica De Caro, Marielisa Alberico, Giovanni Benigni, Terenzio Damiano, Pierluigi Fusco, Angela Iuorio, Giacomo Torretta.
- Ospedale S. Carlo Borromeo – Milano: Milena Racagni, Stefano Muttini.
- ASST della Valle Olona – Busto Arsizio: Girolamo Sala, Paolo Ghiringhelli.
- PO Maria SS. Addolorata – Eboli: Fernando Chiumiento, Laura Baccari.
- Arcispedale Santa Maria Nuova - Reggio Emilia: Enrica Minelli, Boracchia Luca, Federica Bocchi, Francesco Benatti, Jacopo Catellani.
- Ospedale di Belluno - Belluno: Marina Coppola.
- AOU di Ferrara - Ferrara: Alberto Papi.
- Ospedale di Conegliano - Conegliano: Enrico Bosco.
- AO Universitaria Careggi – Firenze: Mnuela Bonizzoli, Chiara Lazzeri.
- Ospedale Della Misericordia - Grosseto: Nencioni Cesira, Camilla Puttini, Tiziana Carli, Leonardo Croci, Marta Corridi.
- Ospedale di Stato di San Marino: Massimo Arlotti, Giulio Guerrini.
- Presidio Ospedaliero Unico AV4 - Fermo: Luisanna Cola, Michela Romanelli.
- AOU Ospedali Riuniti – Ancona: Marina Bonifazi, Stefano Gasparini, Federico Mei, Elisabetta Cerutti.
- AO Ospedali Riuniti - Foggia: Donato Lacedonia.
- Istituto Clinico Humanitas – Rozzano: Armando Santoro, Giacomo Maria Guidelli.
- Ospedale Generale Provinciale – Saronno: Stefano Greco, Andrea Castellan, Gessica Infantino, Laura Camici.
- Ospedale S. M. Annunziata, AUSL Toscana Centro - Grassina: Francesca Covani Frigieri, Vittorio Pavoni
- AOU Senese - Siena: Lucia Migliori, Barbara Rossetti, Francesca Montagnini.
- PO Mauro Scarlato – Scafati: Immacolata Mauro, Elvira Genovese, Antonio Capuozzo, Leonarda Vitiello, Emanuele Sirignano.
- ASST della Franciacorta - Chiari: Paolo Gnesin.
- AOU Federico II – Napoli: Giuseppe Servillo, Alfredo Marinelli.
- AOU di Sassari - Sassari: Daniela Pasero, Lorenzo Casadio, Sergio Babudieri, Giordano Madeddu, Andrea De Vito, Lorenzo Casadio, Melania Ranghitta.
- ASST di Cremona - Cremona: Rodolfo Passalacqua, Fioravanti Antonio, Alfredo Molteni.
- AOU Federico II –Napoli: Ivan Gentile, Antonio Riccardo Buonomo, Riccardo Scotto, Emanuela Zappulo.
- AO S. G. Moscati – Avellino: Giuseppina Dell'Aquila.
- Istituto Clinico S.Anna – Brescia: Angel Bianchetti, Fabio Guerini.
- PO Jazzolino - Vibo Valentia: Alfredo Vallone, Peppino Oppedisano.
- Ospedale Teresa Masselli Mascia Di San Severo - Foggia: Dario Galante.
- Ospedale S. Anna – Como: Luigi Pusterla, Omar Giglio.
- P.O. Maria SS. Addolorata – Eboli: Grazia Russo.
- ASST degli Spedali Civili Di Brescia - Brescia: Enrico Sartori, Cristina Zanardini.
- Ospedale Sen. A. Perrino – Brindisi: Pietro Gatti, Valiani Vincenzo.
- Ospedale Amedeo Di Savoia – Torino: Guido Calleri.
- ASST di Lecco – Lecco: Stefania Piconi, Chiara Molteni.
- ASST di Bergamo Ovest - Bergamo-Treviglio: Giuseppina Dognini.
- Ospedale Castel San Giovanni, Covid Hospital - Piacenza-Castel San Giovanni: Franco Cosimo.
- PO Umberto I - Enna: Luigi Guarneri, Fabrizio Pulvirenti.
- Stabilimento Ospedaliero Castelli – Verbania: Vincenzo Mondino, Gabriella Traballi.
- Ospedale Fatebenefratelli E Oftalmico – Milano: Enrico Iemoli, Antoenlla Grisolia, Riccardo Giorgi, Gabriella Nucera, Valentina Raffaelli, Pietro Marino, Enrica Negro, Lisa Serati, Tamanini Silvia
- AO per l’Emergenza Cannizzaro – Catania: Carmelo Iacobello, Giuseppe Strano.
- Ospedale Sant’Andrea – Vercelli: Lucio Boglione.
- Ospedale Regionale Umberto Parini – Aosta: Alberto Catania, Paola Gipponi.
- AO S.Maria – Terni: Luca Di Cato, Anna Panaccione.
- Policlinico San Marco – Zingonia: Giovanni Vitale, Ilaria Alice Crippa, Matteo Giacomini.
- AO Niguarda Ca Granda – Milano: Adriano Basile, Bellone Andrea.
- Ospedale di Galatina S.Caterina Novella - Galatina: Paolo Tundo.
- Ospedale Versilia – Camaiore: Stefano Buzzigoli, Gerardo Palmiero.
- Ospedale Guglielmo Da Saliceto – Piacenza: Andrea Magnaca, Matteo Silva.
- ASST Lecco – Merate: Massimo Ricci, Stefano Crespi, Bernadetta Pasquino.
- Nuovo Ospedale Di Prato S. Stefano – Prato: Guglielmo Consales.
- Casa Di Cura Privata - Peschiera Del Garda: Damiano Bragantini.
- Ospedale Generale Regionale Miulli, Covid Hospital, Acquaviva Delle Fonti – Bari: Franco Mastroianni, Giulia Righetti, Antonio Scarafino, Michele Bitetto.
- ASST della Valle Olona - Busto Arsizio: Fabio Franzetti.
- Ospedale SS. Trinità – Cagliari: Sandro Piga.
- Ospedale Generale Regionale Miulli, Acquaviva Delle Fonti – Bari: Vito Delmonte
- Ospedale Bisceglie – Bisceglie: Sergio Carbonara, Ruggero Losappio.
- Ospedale Aziendale Di Brunico – Brunico: Christian Dejaco
- Policlinico Umberto I – Roma: Claudio Mastroianni, Gianluca Russo.
- AO S. Croce e Carle – Cuneo: Valerio Del Bono.
- Ospedale Santa Maria Bianca – Mirandola: Fabio Gilioli.
- Ospedale Di Mirano – Mirano: Daniela Barzan, Silvia De Struppi.
- Ospedale Alto Vicentino – Santorso: Antonio Carlotto, Maria Licia Guadagnin.
- AOU di Modena – Modena: Massimo Girardis, Elisabetta Bertellini.
- ASST dei Sette Laghi – Varese: Francesco Dentali.
- PO Sant’Elia – Caltanissetta: Giancarlo Foresta.
- Ospedale Apuane – Massa: Alberto Baratta, Rosangela Viviani.
- ASST Grande Ospedale Metropolitano Niguarda – Milano: Antonio Maria Agrati.
- Istituto Auxologico Italiano - I.S. S.Luca – Milano: Giovanni Battista Perego.
- AOU Policlinico - Vittorio Emanuele – Catania: Arturo Montineri, Rosa Manuele.
- AO Gravina e S. Pietro – Caltagirone: Salvatore Bonfante.
- Nuovo Ospedale Di Prato S. Stefano – Prato: Donatella Aquilini.
- Complesso Ospedaliero A. Cardarelli - Campobasso: Alessandra Prozzo, Donato Santopuoli, Zaira Di Rosa.
- Policlinico San Pietro - Ponte San Pietro: Armando Alborghetti, Paolo Peci, Nikoloz Bakhtadze, Chiara Stefania Pandini, Giovanni Casati, Najat Ashofarir.
- AO Ospedale Niguarda Ca’ Granda - Milano: Giampaolo Casella
- Ospedale Di Trento – Trento: Walter Spagnolli, Silvana Urru, Ivan Marchesoni, Giulia Caminiti
- Istituto Clinica Città di Brescia – Brescia: Elena Argilloni, Elisabetta Danieli, Gianluca Ghirardi, Chiara Maria Antonioli, Alessio Lipari, Paola Zavarise, Francesco Kokaly.
- AOUI Verona Borgo Trento – Verona: Enrico Polati, Leonardo Gottin
- Presidio Ospedaliero Di Vittorio Veneto - Vittorio Veneto: Paolo Lucernoni, Fabio De Conti, Elisabetta Marcon.
- Ente Ospedaliero Ospedali Galliera – Genova: Emanuele Pontali, Elisabetta Blasi Vacca, Carolina Saffioti, Alessia Zunino.
- AOU Ospedali Riuniti di Trieste – Trieste: Erik Roman Pognuz, Giorgio Berlot.
- Ospedale Moscati - Taranto/Statte: Martino Saltori.
- Ospedale Di San Bonifacio - San Bonifacio (VR): Andrea Tedesco.
- ASST del Garda – Desenzano: Silvia Amadasi.
- Ospedale di Treviso –Treviso: Carlo Agostini.
- Ospedale Maggiore – Modica: Maria Antonietta Di Rosolini, Francesco Marino.
- Istituto di Cura Città Di Pavia – Pavia: Guido Bellinzona.
- Ospedale Carlo Urbani Di Jesi – Jesi: Walter Grassi, Marco Di Carlo.
- Ospedale Maggiore Modica – Modica: Guglielmo Scimonello.
- ASST Grande Ospedale Metropolitano Niguarda – Milano: Sandra Nonini, Michele Mondino.
- Villa Maria Cecilia Hospital - Cotignola (RA): Lorenzo Filippo Mantovani, Elena Tenti.
- ARNAS Garibaldi – Catania: Concetta Maria Gabriella Tropea, Daniela Emanuela Di Stefano.
- Ospedale Privato Villalba Hospital: Paolo Guelfi.
- IRCCS S. Raffaele – Milano: Lorenzo Dagna.
- PO Gravina e S. Pietro – Caltagirone: Gianfranco Morgana.
- ASST Grande Ospedale Metropolitano Niguarda – Milano: Lidia Montemurro.
- AOUI Verona - Borgo Roma – Verona: Domenico Girelli, Ernesto Crisafulli, Alessio Maroccia.
- ASST Grande Ospedale Metropolitano Niguarda – Milano: Alessandra Maria Cemuschi.
- Ospedale Gagliardi – Trecenta: Mara Bernasconi.
- Ospedale Maggiore SS. Annunziata – Savigliano: Ugo Zummo.

**TOCIVID-19 Study Coordinators**

- Istituto Nazionale Tumori, IRCCS, Fondazione G. Pascale, Napoli - Clinical Trials Unit: Valentina Barbato, Simona Bevilacqua, Gaetano Buonfanti, Giuliana Canzanella, Giovanni De Matteis, Manuela Florio, Marilena Martino, Maria Teresa Ribecco, Fiorella Romano, Alfonso Savio, Lucia Sparavigna: Melanoma And Cancer Immunotherapy And Developmental Therapeutics Unit: Marcello Curvietto.
- IRCCS Policlinico San Donato – Milano: Michele Citarella.
- ASST Papa Giovanni XXIII – Bergamo: Leonardo Alborghetti.
- Humanitas Gavazzeni – Bergamo: Valeria Nava, Paola Maggioni, Marta Magni.
- AORN Dei Colli – Napoli: Chiara Iommelli, Antonella Bianco.
- Arcispedale Santa Maria Nuova IRCCS Di Reggio Emilia: Romina Corsini, Linda Valli, Maria Paola Ruggieri.
- ASST di Cremona - Cremona: Annalisa Mancini.
- Azienda Ospedaliera San Salvatore – Pesaro: Tiziana Melica.
- Policlinico S. Matteo – Pavia: Alessandra Ferrari, Daniela Cicognini, Mariangela Delliponti, Andrea Zuccarini, Silvia Ciani.
- AOU di Parma - Parma: Davide Raffaeli
- Ospedale Morgagni Pierantoni – Forlì: Luca Donati.
- ASST Santi Paolo e Carlo – Milano: Stefania Cannizzo.
- Ospedale Civile Guastalla – Guastalla: Stefania Lui.
- Arcispedale Santa Maria Nuova IRCCS di Reggio Emilia – Reggio Emilia: Romina Corsini, Linda Valli, Maria Paola Ruggieri.
- Ospedale Infermi Rimini: Luca Santini.
- AOU di Modena – Modena: Enrica Roncaglia, Pasquale Mighali.
- Ospedale Aziendale Di Bressanone – Bressanone: Frederik Eisendle.
- Ospedale San Jacopo – USL Toscana Centro: Giulia Cerino
- Ospedale Guglielmo Da Saliceto – Piacenza: Chiara Citterio, Camilla Di Nunzio.
- ASST di Cremona: Annalisa Mancini.
- Policlinico Universitario A.Gemelli – Roma: Silvia Lamonica.
- Ospedale Sacro Cuore Don Calabria - Negrar (Verona): Silvia Resimini.
- Ospedale Sant’ Andrea - La Spezia: Giovanni Sarteschi.
- Ospedale Di Belluno: Chiara Pavei.
- AOU di Ferrara – Ferrara: Nicholas Battistini.
- Ospedale Generale Provinciale – Saronno: Erika Gazzola.
- ASST di Cremona – Cremona: Annalisa Mancini.
- PO Jazzolino - Vibo Valentia: Marco Miceli.
- ASST di Lecco – Lecco: Silvia Pontiggia.
- ASST di Bergamo Ovest - Bergamo-Treviglio: Veronica Lonati.
- Ospedale Regionale Miulli, Covid Hospital, Acquaviva Delle Fonti – Bari: Giusy Giannandrea.
- Ospedale Maggiore – Modica: Claudio Sortino.
- AOUI di Verona - Borgo Roma – Verona: Serena Ravani.
- Ospedale Delmati – Sant’Angelo Lodigiano: Cristiano Uggeri.
- Ospedale Regionale Umberto Parini – Aosta: Genny Jocollé, Cristina Baré.

**TOCIVID-19 Research Nurses**

- IRCCS Policlinico San Donato – Milano: Irene Baroni, Daniele De Candia, Barbara Fiorini, Katiuscia Chierico, Francesca Romeo, Roberta Bottega, Laura Boccasile, Annamaria Corsaro.
- Azienda Ospedaliera San Salvatore – Pesaro: Claudia Spadoni.
- Ospedale Bassini - Cinisello Balsamo: Ria E Dipartimento Medico Ospedali Di Sesto San Giovanni E Cinisello Balsamo.
- ASST Spedali Civili Di Brescia: Silvia Chiari.
- Azienda Ospedaliera S. G. Moscati – Avellino: Giovanna Ercolino.
- Ospedale F. Spaziani – Frosinone: Vanessa Dell'uomo, Sabrina Viri.
- Ospedale Ca’ Foncello – Treviso: Milena Minato, Lisa Gazzola, Balan Dorina.
- Ospedale Generale Provinciale – Saronno: Davide Gianelli.
- ASST Franciacorta - Presidio Ospedaliero di Chiari: Sonia Maspero.
- ASST della Valle Olona - Busto Arsizio: Maddalena Farinazzo.
- INMI L. Spallanzani, IRCCS – Roma: Paola Zanini, Antonella Sangiovanni.

**TOCIVID-19 Pharmacists**

- Istituto Nazionale Tumori, IRCCS, Fondazione G. Pascale, Napoli - Clinical Trials Unit: Antonia Del Giudice.
- IRCCS Policlinico San Donato – Milano: Maria Margherita Dragonetti, Susanna Bordignon.
- ASST di Cremona – Cremona: Andrea Marco Machiavelli, Giulia Chiodelli, Annalisa Mancini.
- AORN Dei Colli – Napoli: Micaela Spatarella.
- Ospedale Bassini - Cinisello Balsamo: Davide Zenoni, Flavio Niccolò Beretta.
- Ospedale Bellaria – Bologna: Giuseppina Santilli.
- PO Sant’Elia – Caltanissetta: Rita Badagliacca.
- AOU Careggi – Firenze: Manuela Angileri.
- Azienda Ospedaliera S.G. Moscati – Avellino: Luciana Giannelli.
- Ospedale Di Trento – Trento: Annalisa Campomori.
- Ospedale Magalini - Villafranca di Verona: Patrizia Maimone.
- Ospedale Regionale Umberto Parini – Aosta: Andrea Fadda.
- Ospedali Riuniti Padova Sud - Padova: Sonia Faoro
- AOU “Maggiore della Carità” - Novara: Alessia Pisterna

**Other TOCIVID-19 Investigators (centres enrolling patients after March 24, 2020 only)**

- Nuovo Ospedale Garibaldi Nesima – Catania: Bruno Cacopardo, Andrea Marino, Alessio Pampaloni, Benedetto Maurizio Celesia.
- AOU – Foggia: Gilda Cinnella, Daniela Labella, Rosa Roberta Caporusso.
- Ospedale Magalini - Villafranca Di Verona; Maria Danzi, Marta Fiscon, Marina Malena, Doris Fendt, Stefano Nardi.
- AO SS. Antonio e Biagio Cesare e Arrigo: Paolo Stobbione, Maria Laura Savi.
- ASST Melegnano e della Martesana: Andrea De Monte, Alberto Scala, Nicola Lucio Liberato.
- Ospedale Apuane – Massa: Dr. Sauro Luchi; Sauro Luchi, Antonella Vincenti.
- AO Ospedale di Circolo e Fondazione Macchi –Varese: Luca Cabrini.
- AOU di Modena: Giovanni Pinelli.
- AOU di Modena: Lucio Brugioni
- Ospedale Brindisi Senatore. A. Perrino – Brindisi: Domenico Potenza.
- Ospedale S. Maria delle Grazie di Pozzuoli – Napoli: Fabio Giuliano Numis, Giovanni Porta, Maria D'amico, Bianca Iengo.
- AOU Consorziale Policlinico Bari: Gioacchino Angarano, Annalisa Saracino.
- ARNAS Ospedale Civico di Cristina Benfratelli – Palermo: Livio Blasi.
- Ospedale San Giuliano – Giugliano in Campania: Pasquale De Negri.
- Ospedale Di Fermo – Fermo: Stefano Angelici, Antonella Farina, Giuseppe Pio Martino, Giuseppina Bitti.
- Ospedale Bolognini – Seriate: Alberto Tedeschi, Simona De Ponti.
- Ospedale Civile Spirito Santo - Pesacara: Adriana Agostinone, Giustino Parruti, Augusta Consorte, Antonella Frattari.
- AO San Giovanni di Dio e Ruggi d’Aragona- Salerno: Amelia Filippelli, Pasquale Pagliano, Alfonso Masullo, Carmine Sellitto.
- Ospedale Maggiore Carlo Alberto Pizzardi – Bologna: Massimo Reta, Nicolò Rossi, Luigi Raumer.
- Ospedale S. Maria Della Misericordia – Urbino: Silvia Andreassi, Paolo Brancaleoni.
- Ospedale San Francesco – Nuoro: Antonina Carai, Anna Maria Salerno.
- Ospedale Civile S. Salvatore – L’Aquila: Franco Marinangeli, Roberta Mariani, Antonello Ciccone.
- Ospedale Morelli - Sondalo: Carlo Meschini, Gianluca Santoboni, Claudio Angrisani, David Micarelli, Giovanna Tarquini, Vittorio Fregoni.
- AOU Di Ferrara: Carlo Alberto Volta.
- Ospedale INRCA – Ancona: Antonio Cherubini, Maria Simona Del Prete, Erika Ciarrochi.
- Ospedale Di Montebelluna: Francesca Tasca, Andrea Ballarin, Andrea Bianchin.
- Ospedale A. Cardarelli – Campobasso: Romeo Flocco,Vincenzo Cuzzone.
- Azienda Ospedaliera Umberto I –Siracusa: Maurilio Carpinteri.
- Ospedale Di Belcolle - Viterbo: Carlo Meschini, Gianluca Santoboni, Claudio Angrisani, David Micarelli, Giovanna Tarquini.
- Istituto Clinico Beato Matteo Pietro – Vigevano: Pietro Gallotti.
- Presidio Ospedaliero Ospedale Del Mare - Napoli: Federica Torre, Pio Zannetti.
- AO S.M.A. Sede Di Pordenone: Massimo Crapis, Sergio Venturini
- AO Santa Maria Nuova – Firenze: Massimo Barattini, Gianluca Gori.
- PO Annunziata –Cosenza: Antonio Mastroianni.
- Azienda Ospedaliera Regionale S. Carlo – Potenza: Giulio De Stefano, Michele Gilio.
- Ospedale S. Camillo De Lellis - Rieti: Giuseppe Rapisarda, Leonardo Gulisano, Maria Luisa Granata, Sebastiana Saglimbene, Maria Teresa Montalto, Ilaria Grasso, Silvana De Luca, Gaetano Magro, Florinda Messina.
- Ospedale Civile Di Ivrea: Bruno Scapino, Paolo Abrate, Chiara Francisco.
- Ospedale S. Luca - Vallo della Lucania: Laura Pesce.
- PO S. Marta e S.Venera – Acireale: Giuseppe Rapisarda.
- Ospedale Martini – Torino: Mauro Navarra.
- ASST di Vimercate: Michele Agosti, Silvia Pagani, Martina Piluso, Aristodemo Ricioppo.
- Ospedale Per Acuti Mater Salutis - Legnano: Silvia Tognella, Pierangelo Rovere, Marcello Vincenzi, Leonardo Ghirardi.
- ASST di Cremona: Daniele Generali.
- PO Mauro Scarlato –Scafati: Marco Ingrosso, Emilia Desiderio, Raffaele Molaro, Silvio Vitiello.
- Ospedale Maggiore – Chieri: Alessandro Mastroianni, Luca Lancione, Tonia Celeste Paone, Alessandro Meli, Stefano Mainardi, Valentina Rastellino, Antonia Ursillo, Paola di Grigoli, Elena Bovetto, Ivana Marina Stefanetto, Francesca Mazzola.
- Ospedale Ss Antonio E Margherita - Covid Hospital – Tortona: Antonio Daniele, Claudia Bisio, Pietro Delnero, Giovanni Morando, Antonella Nava, Lemut Francesco.
- PO Gorizia e Monfalcone - Gorizia: Fabio Fiammengo, Monica Regis.
- Ospedale San Giovanni Bosco – Torino: Dario Roccatello.
- Ospedale Sen. A. Perrino – Brindisi: Eugenio Sabato.
- Ospedale Civico – Chivasso: Marco Maria Liccardi, Cristina Bretto, Lorenzo Lutri, Enzo Castenetto
- Presidi Ospedalieri Riuniti ASL.6 Ciriè – Torino: Giuseppe Roberti, Maria Francesca Guidi
- ASST Rhodense – Rho: Francesco Bini.
- Ospedale Sandro Pertini – Roma: Maria Crisitna Zappa, Tiziana Trequattrini, Rosario Rivitti, Rossana Vigliarolo, Angela Succu, Marianna Lilli, Mattia Serao, Giuseppina Giogré, Annamaria Ruggieri, Kristoffer Flores, Giuseppe Vairo, Roberto Satira.
- Ospedale degli Infermi – Biella: Anna Lingua.
- Ospedale San Giuseppe – Empoli: Rosario Spina.
- Istituto Nazionale Malattie Infettive INMI L. Spallanzani IRCCS – Roma: Emanuele Nicastri, Gaetano Maffongelli, Filippo Barreca.
- AOU Senese –Siena: Sabino Scollet, Federico Franchi, Camilla Fabbri.
- Ospedale Policlinico – Verona: Pietro Minuz, Andrea Dalbeni.
- AOU Integrata – Verona: Paolo Zanatta, Domenico Gelormini.
- Presidio Ospedaliero V. Buzzi – Milano: Anna Mandelli, Feliciano Galderisi, Elena Zoia.
- Ospedale di Schiavonia: Maria Rita Marchi, Naile De Almeida Neves.
- Presidio Sanitario Gradenigo: Giorgio Carbone.
- PO Boscotrecase: Emilio Di Caterino, Anna Petrone.
- Ospedale S.S. Annunziata – Sassari: Carlo Andrea Usai, Francesco Bandiera.
- Ospedale Civile Di San Candido: Roberto Monti, Alex Hofer.
- AOU Policlinico Vittorio Emanuele – Catania: Giacomo Castiglione.
- Ospedale Mazzini –Teramo: Chiara Angeletti.
- Ospedale Ca’ Granda Niguarda – Milano: Paolo Tarsia
- Ospedale Maggiore – Chieri: Lorenzo Veronese, Paola Daniela Artoni.
- Ospedale San Pietro Fatebenefratelli – Roma: Dora Larussa.
- AO Ospedale Niguarda Ca Granda – Milano: Roberto Fumagalli, Paolo Brioschi.
- Ospedale Santa Croce – Moncalieri: Alessandro Cerutti, Paola Pasquino, Fiore Gilberto.
- Presidio Ospedaliero Aziendale – Fidenza: Luca Cantadori.
- ASST Spedali Civili – Brescia: Gabriele Tomasoni, Lina Rachele Tomasoni.
- Azienda Universitaria Policlinico – Napoli: Nicola Coppola.
- Ospedale di Borgo San Lorenzo: Stefano Spolveri, Costanza Pollastri, Lorenzo Fico
- Madonna Del Soccorso - S. Benedetto:Tiziana Principi, Silvia Pierantozzi.
- AO S.Giovanni/Addolorata – Roma: Col. Costantino Fontana
- PO S. Francesco d’Assisi - Oliveto Citra: Giuseppe Lubrano.
- Presidio Ospedaliero Alto Tevere – Città di Castello: Laura Martinelli, Stefano Bravi
- AOU di Padova – Padova: Paolo Navalesi, Eugenio Serra.
- ASST Franciacorta: Paolo Gnesin, Enrico Cogi.
- PO S. Maria Della Pietà – Nola: Andrea Manzi, Ermenegildo Furino.
- Presidio Ospedaliero Di Chiari: Nicola Dasseni, Claudio Gentilini.
- Ospedale B. Ramazzini – Carpi: Elisa Benatti, Andrea Pignatti.
- Ospedale Civico - Partinico: Giuseppe Aiello, Mario Milia.
- AO Villa Scassi – Genova Sampierdarena: Maria Grazia Covesnon, Annalisa Brianti, Claudio Francesco.
- Ospedale Di Mondovì CN1- Cuneo: Blangetti Ilaria.
- Ospedale Civico – Chivasso: Fiammetta Pagnozzi, Sabrina Mietta.
- Ospedale Pesenti Fenaroli - Alzano: Alberto Rossi
- Ospedale S. Antonio Abate – Gallarate: Lorenzo Maroni, Vittorio Borroni, Claudio Bellintani.
- Ospedale AULSS 15 Alta Padovana: Camilla Sgarabotto, Giada Bizzotto.
- AO.Sant Anna E San Sebastiano – Caserta: Lucio Bucci.
- AO Loreto Mare – Napoli: Giovanni Spagnuolo.
- Ospedale Di Montebelluna: Moreno Agostini, Federico Carlo Caria, Filippo Testa.
- IRCCS AOU S. Martino – Genova: Raffaele De Palma, Giuseppe Murdaca.
- Presidio Ospedaliero di Chiari: Gabriele Zanolini, Nadia Sala.
- Ospedale Del Delta – Lagosanto: Erminio Righini.
- IRCCS AOU S. Martino – Genova: Roberto Pontremoli.
- Ospedale Moriggia Pelascini – Gravedona: Gianmarco Aondio.
- AORN A. Cardarelli – Napoli: Ferdinando Riccardi; Ferdinando Riccardi, Maria Giovanna De Cristoforo, Fausto De Michele.
- Azienda Ospedaliera S. G. Moscati - Avellino: Angelo Storti, Luciana Giannelli.
- PO SS. Trinità – Cagliari: Roberto Perra, Silvia Deidda.
- Azienda Ospedaliera Villa Scassi – Genova Sampierdarena: Caviglia Enrica, Federico Valastro
- ASST di Cremona: Matteo Giorgi Pierfranceschi, Fabio De Gennaro, Anna Laura Nardecchia, Alfredo Molteni.
- ASST di Cremona; Mariateresa Castellini.
- Ospedale San Pellegrino – Castiglione: Giovanni Buetto.
- Casa Di Cura Policlinico – Monza: Giovanbattista Ippoliti.
- Presidio Ospedaliero Di Arco: Domenico Sicheri.
- Presidio Ospedaliero Oglio Po – Casalmaggiore: Maria Grazia Bottoli.
- Ospedale di Vittorio Veneto: Blanca Martinez Lopez De Arroyabe, Alessandra Versaci.
- Casa Di Cura Villa Pini Sanatrix – Civitanova Marche: Giada Pallotti.
- Ospedale Civile E. Agnelli - Pinerolo: Marina Civita, Michele Grio, Nicola Liuzzi, Paola Molino, Mauro Pastorelli, Alberto Ricchiardi, Ferdinando Varbella, Angela Daniela Zeme.
- Ospedale Madre Giuseppina Vannini – Roma: Cinzia Sighieri, Grazia Portale.
- Casa Di Cura Villa Gemma - Gardone Riviera: Alessandro Olivetti, Carlo Pagnoni, Greta Moschini, Sabrina Boni, Alberto Guerra, Roberta Scudellari, Sabrina Vella.
- Presidio Ospedaliero Di Borgo Valsugana: Sandro Inchiostro.
- PO S. Maria dell’Olmo – Cava de Tirreni: Ornella Piazza.
- IRCCS Multimedica – Milano: Salvatore Guarino, Giorgio Aldegheri.
- PO Paolo Borsellino – Marsala: Giovanna Napoli.
- AOU Careggi – Firenze: Alessandro Morettini, Eleonora Caldini, Lorenzo Menicacci.
- AOU Careggi – Firenze: Filippo Pieralli, Monica Torrini.
- AOU – Careggi – Firenze: Loredana Poggesi.
- Clinica Pinna Pintor Srl – Torino: Enrico Maria Visetti.
- Policlinico di Alessandria: Enrico Maria Visetti.
- Presidio Ospedaliero Riuniti – Reggio Calabria: Carmelo Mangano.
- Casa Di Cura Villa Barbarano - Salo: Stefano Visconti.
- ASST di Bergamo Est: Pasquale Maietta.
- Ospedale SS. Capitanio e Gerosa – Lovere: Elisa Banfi, Stefania Cartella.
- Ospedale F. Spaziani – Frosinone: Bruna Venturi, Antonia Nuceri.
- Ospedale San Pellegrino - Castiglione: Elena Chiesa, Enrico Pacentra.
- Ospedale San Pellegrino - Castiglione: Gianluigi Panzolato.
- AO Villa Scassi – Genova Sampierdarena: Michella Giannotti, Cristina Bianchi.
- AOU di Modena: Antonello Pietrangelo.
- AOU Careggi – Firenze: Ombretta Para, Maria Serena Rutili.
- Presidio Sanitario Ospedale Cottolengo - Torino: Roberto Russo, Maurizio Lanfranco, Elisa Scalabrino.
- AOU Sant’Andrea – Roma: Agostino Tafuri.
- **Other TOCIVID-19 Study Coordinators (centres enrolling patients after March 24, 2020 only)**
- Ospedale degli Infermi – Biella: Elisa Perfetti.
- AO Villa Scassi – Genova Sampierdarena: Tosca Chiarello, Cristina Bianchi.
- Ospedale Di Montebelluna: Luca Cancanelli.
- AORN A.Cardarelli – Napoli: Manuela Otero.
- Casa Di Cura Villa Gemma - Gardone Riviera: Sabrina Vella, Greta Pannella, Francesco Bellucci.
- Presidio Sanitario Ospedale Cottolengo – Torino: Giovanna Ferrero.
- AOU Sant’Andrea – Roma: Carmen Vico.
- Ospedale Civile Spirito Santo - Pesacara: Maria Serafina Stillante.

**Other TOCIVID-19 Research Nurses (centres enrolling patients after March 24, 2020 only)**

- Ospedale A.Cardarelli – Campobasso: Giovanna D'Andrea.
- Ospedale S.Camillo De Lellis Rieti: Filippo Amoroso, Antonio Arcidiacono, Anna Maria Bella, Agata Belsito, Ylenia Berté, Giulia Carubia, Maria Grazia Caruso, Orazio Casella, Francesco Chiereleson, Chiara Costa, Daniela De Franco, Giuseppe Germanà, Antonio Messina, Diana Musumeci, Concetta Noto, Marco Valenti.
- PO Mauro Scarlato - Scafati: Carlo Sorrentino, Rosanna Panico, Giuseppe Schettino, Jolanda Piccoli, Antonio Pepe, Francesco De Rosa, Mario Ottaviano, Gerarda Marrazzo.
- Ospedale Sandro Pertini – Roma: Gianna Raponi, Stefania Diberardino.
- AOU Careggi – Firenze: Simona Bausi.
- Presidio Sanitario Ospedale Cottolengo – Torino: Alessandro Ferrari.

**Other TOCIVID-19 Pharmacists (centres enrolling patients after March 24, 2020 only)**

- Ospedale SS Antonio E Margherita, Covid Hospital – Tortona: Sara Francesca Marini.
- Presidio Sanitario Gradenigo: Elena Giubellino, Giorgio Innocenti.
- AORN A. Cardarelli – Napoli: Gaspare Gugliemi.
- Ospedale di Vittorio Veneto: Daniela Maccari, Izabela Baciu.
- Ospedale Maggiore – Chieri: Tonia Celeste Paone.
